# Molecular Details of Actinomycin D-Treated MRSA Revealed via High-Dimensional Data

**DOI:** 10.3390/md20020114

**Published:** 2022-01-31

**Authors:** Xuewei Xia, Jun Liu, Li Huang, Xiaoyong Zhang, Yunqin Deng, Fengming Li, Zhiyuan Liu, Riming Huang

**Affiliations:** 1 Guangdong Provincial Key Laboratory of Food Quality and Safety, College of Food Science, South China Agricultural University, Guangzhou 510642, China; xiaxuewei@stu.scau.edu.cn (X.X.); huangli@stu.scau.edu.cn (L.H.); dengyq0811@163.com (Y.D.); lifengming@hzu.edu.cn (F.L.); aiden@stu.scau.edu.cn (Z.L.); 2 Laboratory of Pathogenic Biology, Guangdong Medical University, Zhanjiang 524023, China; lj2388240@gdmu.edu.cn; 3 Joint Laboratory of Guangdong Province and Hong Kong Region on Marine Bioresource Conservation and Exploitation, College of Marine Sciences, South China Agricultural University, Guangzhou 510642, China; zhangxiaoyong@scau.edu.cn

**Keywords:** methicillin-resistant *Staphylococcus aureus*, actinomycin D, *Streptomyces parvulus*, mechanism

## Abstract

Methicillin-resistant *Staphylococcus aureus* (MRSA) is highly concerning as a principal infection pathogen. The investigation of higher effective natural anti-MRSA agents from marine *Streptomyces parvulus* has led to the isolation of actinomycin D, that showed potential anti-MRSA activity with MIC and MBC values of 1 and 8 μg/mL, respectively. Proteomics-metabolomics analysis further demonstrated a total of 261 differential proteins and 144 differential metabolites induced by actinomycin D in MRSA, and the co-mapped correlation network of omics, indicated that actinomycin D induced the metabolism pathway of producing the antibiotic sensitivity in MRSA. Furthermore, the mRNA expression levels of the genes *acnA*, *ebpS*, *clfA*, *icd*, and *gpmA* related to the key differential proteins were down-regulated measured by qRT-PCR. Molecular docking predicted that actinomycin D was bound to the targets of the two key differential proteins AcnA and Icd by hydrogen bonds and interacted with multiple amino acid residues of the proteins. Thus, these findings will provide a basic understanding to further investigation of actinomycin D as a potential anti-MRSA agent.

## 1. Introduction

Antimicrobial resistance in microbial pathogens is an important health problem, as infections caused by drug-resistant bacteria increase the death rate every year all over the world [1,2]. Methicillin-resistant *Staphylococcus aureus* (MRSA) could cause many kinds of infections, such as skin abscess, endocarditis, joint infections, and necrotizing pneumonia. MRSA used to be mainly found in the hospital environment but has now also emerged in the community environment [3]. In the hospital environment, there are 60–70% *S. aureus* infections caused by MRSA, which causes the most significant number of spreading infections in all drug-resistant bacteria [4]. While highly effective therapeutic drugs, such as linezolid, daptomycin (lipopeptide), and Synercid^®^, are being applied to inhibit the pathogens, some reports indicated that these bacteria also show emerging resistance to these drugs [5]. Although the resistance of each new antibiotic eventually comes out within a short year time after its promotion, it is necessary to search for novel natural antibacterial agents to inhibit antibiotic-resistant bacteria of pathogenic microorganisms [6].

Recent research studies in medical science have triggered discovery of the novel natural antimicrobial agents from numerous microorganisms [7]. Among these microbes, especially, the genus *Streptomyces* is one of the most important resources of searching for new antimicrobial agents because of its vital producers of new natural pharmaceutical precursors with diverse structural properties [8,9]. Approximately 67% of the natural therapeutic antibiotics originated from the genus *Streptomyces*, and there still are many potent natural isolates that need to be further explored [10]. Actinomycins are a major class of antibiotics in *Streptomyces*, among which actinomycin D is one of the most widely studied [11]. Actinomycin D has good antitumor activity and has been used as a clinical antitumor anticancer drug [12]. As an antibiotic, Actinomycin D and its analogs also have antibacterial activity against a variety of bacteria, most of which are Gram-positive, such as *S. aureus*, *S. epidermidis*, *Enterococcus faecalis*, with an exception for *Xanthomonas oryzae* being Gram-negative [13,14,15]. Moreover, it is reported that drug-resistant strains of the aforementioned Gram-positive bacteria also show similar actinomycin D susceptibility to their susceptible bacteria. Wang et al. [12] isolated actinomycin D from the marine-derived actinomycete *Streptomyces* sp. IMB094, which had a better antibacterial effect with MIC of 0.125–0.5 μg/mL on MRSA, methicillin-resistant *S. epidermidis*, and vancomycin-resistant *Enterococcus*.

In the process of our searching for new natural anti-MRSA precursors, a marine antibacterial *Streptomyces parvulus* extract showed a stronger anti-MRSA effect. Our previous investigation on the secondary metabolites of *S. parvulus* revealed that actinomycin D was the main secondary metabolites of this strain [16]. Although there have been some studies on the screening of actinomycin D against MRSA, the mechanism remains unclear [12,13]. Thus, it is still necessary to obtain more information on the anti-MRSA mechanism of actinomycin D.

In this work, MRSA as the drug-resistant strains of *S. aureus* were different from the previous research object [16]. On the one hand, the same research route was adopted as before, through proteomics integrated with metabolomics analysis for actinomycin D-treated MRSA to confirm proteins and metabolites with observably changed expression and identify the functions, so as to clarify the inhibition and molecular effect details of actinomycin D to MRSA. On the other hand, different from previous methods, qRT-PCR was used to verify the mRNA expression levels of key differential proteins, and molecular docking was used to predict the docking mode of actinomycin D to key proteins. The effects of antibiotics on bacteria, and even drug-resistant bacteria, involve numerous and complex biomolecular networks, which meet great challenges to research key targets [17]. Omics analysis can provide us with a large information, and molecular docking as predictive biology may allow us to better obtain some key information from a lot of data [18]. These research studies will help to better understand the potential molecular role of actinomycin D on MRSA.

## 2. Results

### 2.1. Effect of Actinomycin D on Bacterial Morphology

**Figure 1:**
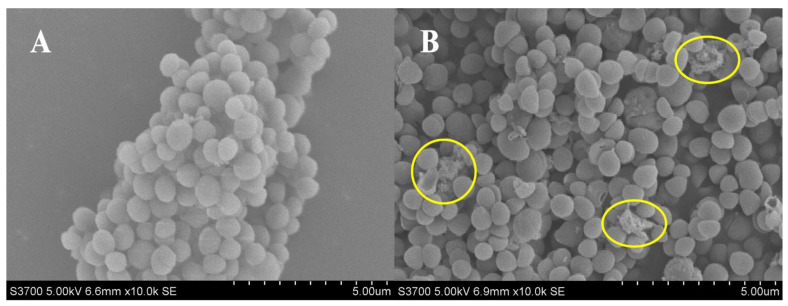
(**A**,**B**) SEM analysis of MRSA treated with and without actinomycin D. (**A**) Control group; (**B**) treatment group.

Actinomycin D showed an obvious anti-MRSA activity, with MIC value of 1 μg/mL and MBC value of 8 μg/mL. Using SEM analysis, changes in the bacterial morphology exposed to actinomycin D were researched. The bacteria cultured without actinomycin D were performed as a control. As revealed in Figure 1, the control group bacteria showed an intact and smooth membrane with normal morphology. After being treated by actinomycin D, bacteria displayed the leaking of bacterial content, causing differences in morphology. Lysed cells and debris were observed in the treatment group.

### 2.2. Effect of Actinomycin D on Protein Profiles

**Figure 2:**
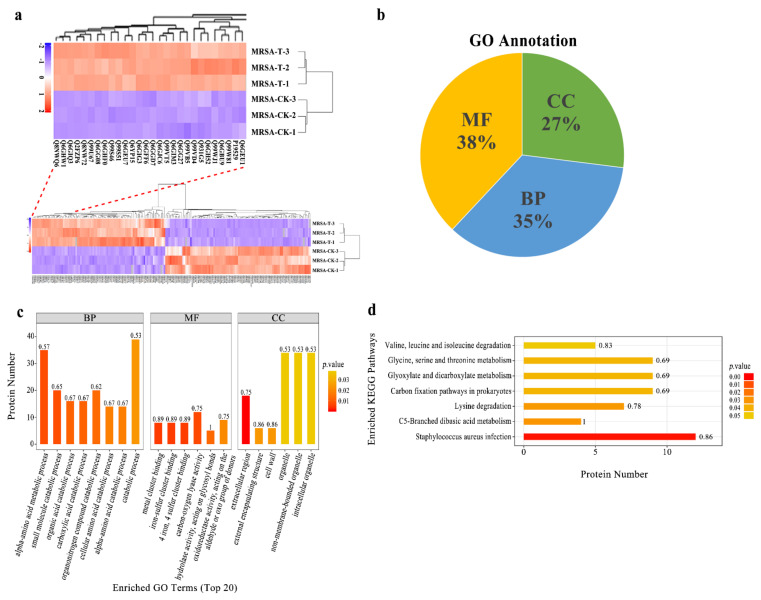
(**a**) Hierarchical cluster analysis for all the DEPs. MRSA-T: treatment group; MRSA-CK: control group. Down-regulated proteins are displayed as purple, and up-regulated proteins are displayed as red. Proteins that newly arising and undetected in the D-T were not embraced. (**b**) GO annotation for the DEPs. BP: biological process, MF: molecular function, CC: cellular component. (**c**) Enriched GO term (top 20). The numbers beside the bar indicated the enrichment factor, and the color of the bar indicates the *p*-value. (**d**) KEGG pathway enrichment of the identified DEPs.

The label-free proteomic analysis method was used to analyze the MRSA affected by actinomycin D. Compared with the control group, 261 differentially expressed proteins (DEPs) were significantly changed in the group treated by actinomycin D screened with log_2_(fold changes) > ± 2 and *p*-value < 0.05, including 95 up-regulation and 166 down-regulation DEPs (Table S1). Among them, 9 proteins were regarded as up-regulated DEPs because they were not detected in the control group and 72 proteins were regarded as down-regulated DEPs because they were not detected in the treatment group. Hierarchical cluster study was conducted for all the identified DEPs of the two groups (Figure 2a). The DEPs in the group treated by actinomycin D did not cluster together as in the control group.

Gene ontology annotation gave an available way to profile the protein properties of the identified proteins. It was used to analyze the functions of the DEPs [19]. Furthermore, GO was categorized into three classifications, including biological process (BP), molecular function (MF), and cellular component (CC). The GO studies showed that the DEPs were sub-classified into 34 hierarchically-structured GO categories (Table S2). Thirty-eight percent DEPs were related to molecular function, 35% DEPs were related to biological process, and 27% DEPs were related to cellular component (Figure 2b). Concerning biological processes, 182 DEPs and 187 DEPs were related to the GO term of metabolic processes and cellular processes, respectively. In addition, the GO terms of biological regulation, response to the stimulus, regulation of the biological process, multi-organism process, cellular component organization or biogenesis, localization, signaling, and developmental processes were also involved. At the molecular function level, 167 DEPs were involved in catalytic activity, and 142 were related to binding. In the cellular component, most of the DEPs were ontologically involved in the function of the cell, cell wall, protein-containing complex, organelle, membrane, extracellular region, membrane part, and organelle part (Figure 2c).

Many proteins are known as vital enzymes in metabolic pathways. The KEGG results showed that the 261 DEPs were involved in 110 signaling pathways in total (Table S3). From these, the top metabolic pathways with *p*-value < 0.05 were *Staphylococcus aureus* infection, C5-branched dibasic acid metabolism, lysine degradation, carbon fixation pathways in prokaryotes, glyoxylate and dicarboxylate metabolism, glycine, serine, and threonine metabolism, and valine, leucine, and isoleucine degradation (Figure 2d). These results indicated that, under actinomycin D-treated MRSA, many DEPs might be the vital enzymes participated in metabolic pathways and performed different functions.

### 2.3. Effect of Actinomycin D on Metabolite Profiles

**Figure 3:**
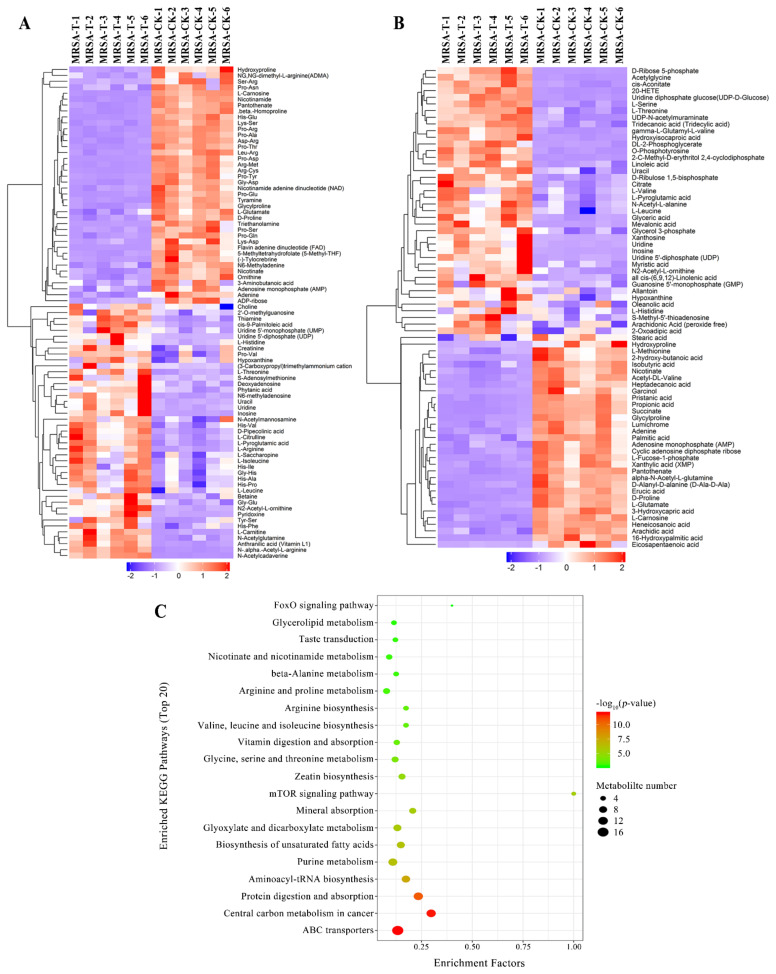
(**A**,**B**) Hierarchical cluster heat map of differential metabolites in positive mode (**A**) and negative mode (**B**). MRSA-T: treatment group; MRSA-CK: control group. Down-regulated proteins are displayed as purple, and up-regulated proteins are displayed as red. (**C**) Pathways encompassed by the differential metabolites (TOP 20).

Given the conditions of an orthogonal partial least-squares discrimination analysis (OPLS-DA) model (*p*-value < 0.05, variable importance in the projection (VIP) > 1), distinctly different metabolites were detected. The hierarchical cluster analysis was used to research changes in candidate metabolic processes and the accurate selection of marker metabolites (Figure 3a,b). At the same time, the relationship between samples and the differences in expression of metabolites in different samples can be more comprehensively and intuitively displayed. As a result, 144 obvious changed metabolites were identified (Table S4). In total, 72 metabolites were down-regulated, while 72 metabolites were up-regulated in the treatment group.

For the analysis of related pathways, the distinctly changed metabolites were uploaded to the KEGG database, and the differentially expressed metabolites were mostly related to the ABC transporters, protein digestion and absorption, aminoacyl-tRNA biosynthesis, central carbon metabolism in cancer, and purine metabolism (Figure 3c). The changes of the metabolites contribute significant information for further research of the changes in the group treated by actinomycin D.

### 2.4. Proteomics-Metabolomics Data Integrated Analysis

**Figure 4:**
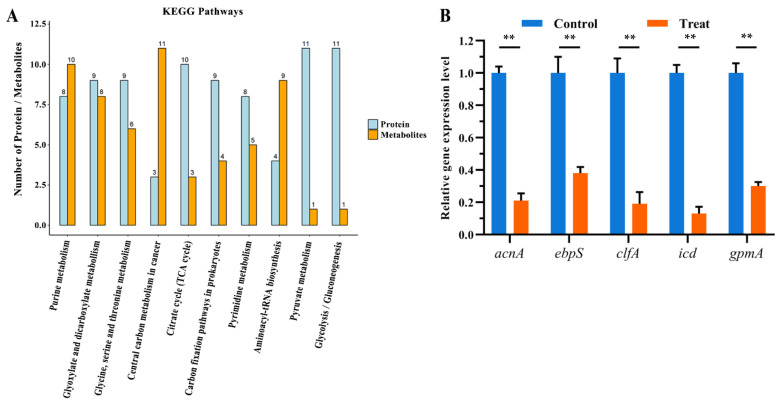
(**A**) Common pathways of vital proteins and metabolites. (**B**) Relative gene expression levels of gene *acnA*, *ebpS*, *clfA*, *icd*, and *gpmA* between the two groups (**, *p* < 0.01).

Based on the changes of proteins and metabolites, metabolic pathways were chosen as the carrier and performed a mapping analysis to combine the data of proteome and metabolome. A total of 66 metabolic pathways showed differences (Table S5). Research studies of these identified metabolic pathways revealed that they were mostly related to amino acid metabolism. Furthermore, we screened the metabolic pathways related to lysine degradation, glycine, serine, and threonine metabolism, and valine, leucine, and isoleucine degradation, among others (Figure 4a).

According to annotation, the significantly changed proteins and metabolites were mainly related to the glycine, serine, and threonine metabolism pathway. The proteins and metabolites that participated in glycine, serine, and threonine metabolism were quite active when treated by actinomycin D, in which 6 metabolites were up-regulated, and 8 proteins were down-regulated.

### 2.5. Validation of Select DEPs by qRT-PCR

To verify the authenticity and accuracy of the proteomics data, the change of proteins was researched at the mRNA level using qRT-PCR. Considering that the DEPs in two groups are referred to many functional categories, we chose 5 genes that participated in TCA cycle, amino acid metabolism, as target genes for qRT-PCR analysis. Compared with the proteomics results, the mRNA expression levels of 5 genes among the target proteins experimented by qRT-PCR suggested coincident changes with the corresponding protein expression levels (Figure 4b). Therefore, the results showed that the proteomics results could be identified in the mRNA level.

### 2.6. Molecular Docking Studies

**Figure 5:**
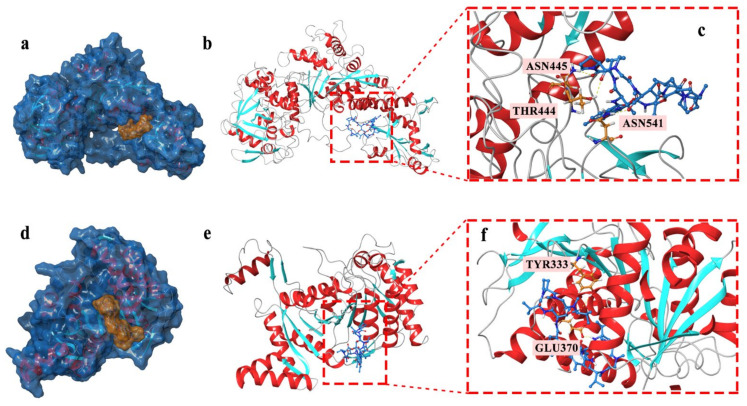
(**a**–**c**) Actinomycin D accommodated in AcnA (**a**); overview of the docked pose (docking score: −5.729) (**b**); hydrogen bond interactions of the actinomycin D with AcnA (**c**). (**d**–**f**) Actinomycin D accommodated in Icd (**d**); overview of the docked pose (docking score: −5.65) (**e**); hydrogen bond interactions of the actinomycin D with Icd (**f**). The proteins models of AcnA and Icd used in molecular docking were homology models.

Based on the docking studies, the location of the hydrogen bond, docking score, and binding energy and ligand-protein pair was predicted and is listed in Table 1. According to the three-dimensional docking mode, we found that actinomycin D might bind to the targets of all three proteins by hydrogen bonds and interact with multiple amino acid residues of the proteins. Three hydrogen bonds were found between actinomycin D and ASN445, THR444, and ASN541 in AcnA as a possible binding way (Figure 5a–c). The predictive binding energy and docking score was calculated as −48.15 kcal/mol and −5.729, respectively. Moreover, actinomycin D might bind to the amino acid residues of Icd located in TRY333 and GLU370 by hydrogen bond, and the predictive binding energy and the docking score were measured as −26.27 kcal/mol and −5.65, individually (Figure 5d–f). In conclusion, hydrogen bonds might be the driving force for the interaction between actinomycin D and the proteins. The results indicated that actinomycin D could be bound to these proteins to inhibit the growth of MRSA.

## 3. Discussion

Proteomics and metabolomics are subsequent additions performing the comprehension of the function and relating to sequential events in omics studies. Proteomics primarily exposes genetic modifications and exhibits the expression spectrum of the proteins containing the research of protein structure. On the contrary, metabolomics shows an intact analysis of the compounds with low molecular weight. It has been reported by Gahlaut et al. [20] that the comprehensive explication of biological responses in different conditions can be written at the level of transcriptomics, proteomics, and metabolomics, and modulations in the metabolome are reflected by proteome. The results show the significance of addressing the consolidation of proteome and metabolome in the current research. Akpunarlieva et al. [21] integrated proteome and metabolome to show us an approach of relating biochemistry to phenotype, further anchorage was furnished to the present study.

### 3.1. Antibiotic Sensitivity of MRSA Affected by Actinomycin D

Glycine, serine, and threonine have an impact on the growth of microbes [22]. It has been reported that glycine, serine, and threonine metabolism is a significant pathway that refers to antibiotic sensitivity. Moreover, according to the research, the synergy of the promoted glycine, serine, and threonine metabolism and glucose can enhance the kanamycin-mediated killing [23]. According to the proteomics and metabolomics results, 9 DEPs (all down-regulated) and 6 differently metabolites (all up-regulated) participated in the glycine, serine, and threonine metabolism pathway. Via the oxidative cleavage of glycine, glycine cleavage system H protein (GcvH) is one of the four proteins that compose the glycine cleavage complex, vital for the synthesis of C_1_ (one-carbon units) for cell metabolism. The abundance of this protein is promoted by exogenous glycine, and is suppressed by the presence of purines [24]. Glycine betaine is significant for the existence of bacteria in various environments [25]. Oxygen-dependent choline dehydrogenase (BetA) and GcvH oxidize choline to betaine aldehyde and subsequently convert to glycine betaine [26]. Interestingly, we found that choline and betaine were up-regulated. Furthermore, the results showed that GcvH and BetA were down-regulated. The down-regulation of GcvH and BetA inhibited the oxidization of choline to betaine aldehyde, and we presumed the protein hydrolysis might result in the up-regulation of betaine. 2,3-Bisphosphoglycerate-dependent phosphoglycerate mutase (GpmA) was down-regulated in the proteomics study; it may motivate the interchange between 2-phosphoglycerate and 3-phosphoglycerate. GpmA functions as a potential target for new antibiotics as a crucial enzyme in energy metabolism and glycolysis [27]. Dihydrolipoyl dehydrogenase (PdhD) involved in glycine, serine, and threonine metabolism is also regarded as vital enzymes for the anaplerosis of the TCA cycle [28]. Therefore, the down-regulation of the PdhD might disturb the TCA cycle. It has been reported that the TCA cycle plays an important role in mediating the biofilm formation of MRSA, specifically by influencing the matrix composition [29]. Moreover, a recent report has demonstrated that the fluctuation of TCA cycle is an antibiotic-resistant mechanism. Our omics studies showed the expression of isocitrate dehydrogenase (NADP) and aconitate hydratase A (AcnA) in the TCA cycle were decreased. AcnA could transform the citrate to cis-aconitate. The decrease of the AcnA would inhibit the conversion to disturb the TCA cycle. The results above indicated that actinomycin D might affect the antibiotic sensitivity of MRSA by disturbing the glycine, serine, and threonine metabolism and TCA cycle.

Furthermore, the bacterial cell wall is an important target of many antibiotics [30]. By scanning electron microscopy analysis, changes in the MRSA morphology were observed. According to the results, MRSA treated by actinomycin D could break the cell and showed a deep degree of abnormal cell shape. These results can be expounded by speculating that the cell wall may be damaged by actinomycin D. Moreover, the purine biosynthetic pathway plays a vital part in cell wall synthesis, and the whole purine biosynthetic pathway was indicated to be in invertible and robust restriction under the cases that the cell wall synthesis was disturbed [31]. The proteomics results in our research showed that the 4 proteins that participated in purine metabolism were up-regulated, and 4 proteins were down-regulated. The metabolomics results showed that 4 metabolites related to purine metabolism were inhibited, and 5 metabolites were stimulated significantly. The disturbance of purine metabolism was influential for the purine content in the cell to cope with the growing need for ATP due to the superfluous cell wall material produced [32]. The results indicated that the purine metabolism pathway was disturbed in MRSA treated by actinomycin D. According to the omics analysis, some proteins involved in cell wall synthesis are down-regulated, including lactonase Drp35 (a 35-kDa drug-responsive protein), clumping factor A (ClfA), and elastin-binding protein of *S. aureus* (EbpS). Drp35 relates to the cell wall synthesis of MRSA, preserving the cell to exist in a serious environment [33]. ClfA, a staphylococcal surface protein, could bind with fibrinogen [34]. The EbpS is an adhesin functional for adhering to host cells by binding with elastin. It has been confirmed that the EbpS in MRSA could regulate zinc-dependent biofilm formation [35]. The down-regulation of these proteins might inhibit the growth of the cell wall to influence the antibiotic sensitivity of MRSA.

Given that oxidative stress is associated with antibiotic treatment, low levels of ROS could be used as an adjuvant to potentiate the antibacterial activity of commercial antibiotics [36]. During aerobic respiration, some reactive oxygen species (ROS) that impair the DNA, protein, and lipids within cells are produced. L-lactate dehydrogenase 1 (Ldh1), which was down-regulated in our study, responsible for catalyzing the conversion of pyruvate to lactate. Since Ldh1 converts NAD+ to NADH in the actions, it plays a vital role in keeping the homeostasis of redox in bacteria [37]. NADPH can reduce glutathione through glutathione reductase, which transforms reactive H_2_O_2_ to H_2_O by glutathione peroxidase. If NADPH is insufficient, the H_2_O_2_ would attack the cell when transformed into hydroxyl free radicals [38]. The down-regulation of glutathione peroxidase showed that H_2_O_2_ transformation was suppressed, which would cause the gathering of H_2_O_2_. Therefore, the increasing H_2_O_2_ during the treatment of MRSA likely produced extra ROS, which might damage the cellular compounds. Glutathione is a vital antioxidant in bacteria and is used to prevent oxidative injury influenced by ROS [39]. Pathway study revealed that the enzymes related to glutathione metabolism were down-regulated, showing that the antioxidant shield system was broken. Moreover, two enzymes involved against ROS, including peroxidase and superoxide dismutase, were down-regulated, but catalase was up-regulated [40]. Based on the carbohydrate metabolism and glutathione metabolism that were restrained, as well as those proteomic data, we speculated that ROS might gather via increasing oxidative stress treated by actinomycin D. The accumulation of ROS results in apoptosis [41]. Thus, the increased oxidative stress during the treatment of MRSA likely produces more ROS that could damage cellular components, containing the cell membrane component, such as unsaturated fatty acids.

### 3.2. Virulence Factor of MRSA Affected by Actinomycin D

The Serine-aspartate repeat-containing protein D (SdrD) protein is vital for MRSA to bind to host cells, enhance the virulence, and exist in the blood [42]. ClfA is regarded as a critical MRSA virulence factor as a result of it having been suggested to increase cell virulence in experimental models of sepsis [43], skin infection [44], septic arthritis [45], and endocarditis [46]. ClfA combines with the dimeric host fibrinogen through the carboxyterminal domain of the fibrinogen gamma chain, causing bacterial aggregation in plasma or purified fibrinogen [47]. The down-regulation of the SdrD and ClfA proteins in the current research indicates that the cells have been suppressed by actinomycin D. Interestingly, the accessory gene regulator A (AgrA) and S-ribosylhomocysteine lyase (LuxS) proteins, respectively, exhibited down-regulation and up-regulation. Both of the proteins related to quorum-sensing have been suggested to play vital parts in many biological processes, containing host infection and biofilm formation [48]. Previous research showed that LuxS regulates virulence factors and plays a role in metabolism [49]. Thus, the different changed types of AgrA and LuxS might be due to their respective involvement in different functional regulations. The result showed that actinomycin D might regulate virulence factors, such as SdrD, ClfA, AgrA, and LuxS, to reduce virulence.

## 4. Materials and Methods

### 4.1. Preparation of Actinomycin D

The preparation of actinomycin D from marine *Streptomyces parvulus* was performed according to the same method used in our previous study [16]. Briefly, strain SCAU-062 was identified as *Streptomyces parvulus* and deposited in the Joint Laboratory of Guangdong Province and Hong Kong Region on Marine Bioresource Conservation and Exploitation, College of Marine Sciences, South China Agricultural University.

The strain SCAU-062 was inoculated and cultivated in 150 replicate 1000 mL Erlenmeyer flasks, each containing 250 mL Luria Bertani (LB) broth (dextrose 1 g/L, yeast extract 5 g/L, peptone 10 g/L, sodium chloride 5 g/L), in a rotary shaker (150 rpm) at 28 °C. Cultivated for 7 days, a total of 30 L of fermentation broths was exhaustively extracted with ethyl acetate (3 times × 30 L) at 25 °C for 3 × 7 days. The solution was evaporated (P = 0.01 MP, T = 45 °C) to get a residue (5 g). The ethyl acetate soluble portion was applied to column chromatography on silica gel by using petroleum ether/acetone (from 7:3 to 0:1) as the eluent, giving eight fractions (A–H). Semi-preparative reversed-phase HPLC further purified fraction C (Waters Prep C18 column, 150 × 19 mm, 5 μm, 2 mL/min) to afford purified actinomycin D (45% MeOH/H_2_O, t_R_ = 24.1 min) that was identified in our previous report [16].

### 4.2. Microorganism and Medium

The strain MRSA ATCC 43300 was acquired from the American Type Culture Collection (ATCC, Manassas, VA, USA). After being incubated on LBA (peptone 10 g/L, sodium chloride 5 g/L, dextrose 1 g/L, yeast extract 5 g/L and agar 15 g/L) for 24 h at 37 °C, the strain MRSR ATCC 43300 was inoculated in 200 mL LB medium and cultivated on a shaker at 150 rpm for 24 h at 37 °C to gain the bacterial suspension.

### 4.3. Minimum Inhibitory Concentration and Minimum Bactericidal Concentration Measurement of MRSA Effected by Actinomycin D

The minimum inhibitory concentration (MIC) of actinomycin D on MRSA was measured by a broth micro-dilution method described by Dellavelle et al. [50]. Briefly, the bacterial suspension (100 μL) of strain MRSA was incubated in Mueller-Hinton (MH) broth and cultivated at 150 rpm at 37 °C. Fifty microliters of different concentrations of actinomycin D (0.25, 0.5, 1, 2, 4, 8, 16, and 32 μg/mL) were added to the bacterial culture when the MRSA grew to logarithmic phase (OD_600_ reached 0.3 to 0.5). For control, a positive control group consisted of 150 μL of MH broth and 50 μL of bacterial culture, and the negative control group contained 200 μL of MH broth only. All groups were cultured at 37 °C for 24 h, and OD was determined at 600 nm. Each treatment was performed in three replicates. The MIC value was calculated as described by Dellavelle et al. [50]. After measuring the MIC, fermented liquid of 100 μL from each well that shows no bacterial growth was streaked onto MH agar plates at 37 °C for 20 h. The value of minimum bactericidal concentration (MBC) was the lowest concentration that kills all of the initial bacterial population, showing no colonies on the MH agar [51].

### 4.4. Scanning Electron Microscopy (SEM) Analysis

To research morphology under treatment of actinomycin D by SEM analysis, MRSA was incubated in 100 mL of LB broth for 24 h with or without actinomycin D treatment. The cells of MRSA were gathered by centrifugation and rinsed thrice with 0.1 M phosphate-buffered saline (PBS) buffer. Then, 2.5% Glutaraldehyde was used to fix the cells for 2 h. The rest of the process was conducted according to the method of Suo et al. [52]. The morphology of the bacteria was photographed by a Hitachi s-3700n tungsten filament SEM (Hitachi, Tokyo, Japan).

### 4.5. Label-Free Quantitative Proteomics

The label-free quantitative proteomics analysis was performed by the previously described methods [28]. The measurements were carried out in sextuplicate.

#### 4.5.1. Sample Preparation

The MRSA was harvested by centrifugation (14,000 rpm, 3 min) and rinsed thrice with PBS buffer. The cells were processed for total protein extraction. The protein concentration was measured by BCA Protein Assay Kit (Bio-Rad, Hercules, CA, USA). Then, 250 μg protein of each sample was conducted by the reported Filter-aided sample preparation (FASP Digestion). A UV light spectral was used to estimate the peptide concentration density at 280 nm.

#### 4.5.2. Liquid Chromatography (LC)-Electrospray Lionization (ESI) Tandem MS (MS/MS) Analysis

C_18_ Cartridges (Sigma-Aldrich, Saint Louis, MO, USA) were used to desalt peptides in every sample, and, finally, 0.1% (*v*/*v*) trifluoroacetic acid (40 μL) was added to reconstitute. MS experiments were conducted on a QExactive mass spectrometer (Thermo Fisher Scientific, Waltham, MA, USA). Those containing peptide (5 μg) of buffer A included 0.1% formic acid and 2% acetonitrile, separated with a gradient of buffer B including 0.1% formic acid and 80% acetonitrile, conducted by a C_18_ reversed-phase column (Thermo Scientific Easy Column, Waltham, MA, USA) at a speed of 250 nL/min regulated using IntelliFlow technology for 120 min. The measure of the target value was based on predictive Automatic Gain Control (pAGC). Every sample was performed in MS experiments thrice.

#### 4.5.3. Sequence Database Search and Data Analysis

MaxQuant software version 1.3.0.5 (Max Planck Institute of Biochemistry, Munich, Germany) was used to conduct the MS data, and the data was retrieved in the UniProtKB *Staphylococcus aureus* database. Label-free quantification was conducted on MaxQuant as previous studies reported [53]. Software Origin version 6.1 (OriginLab, Northampton, MA, USA) was used to perform statistical analysis. The DEPs were selected by the following conditions: log_2_(fold changes) > ± 2, and *p*-value < 0.05.

### 4.6. Untargeted Metabolomics Methods

#### 4.6.1. Sample Collection and Preparation

The measurements were performed in triplicate. Four hundred microliters of acetonitrile/methanol (1:1, *v*/*v*, 4 °C) were mixed with 100 μL aliquots to eliminate the protein of the samples at 4 °C. The mixture was centrifuged (14,000 *g*, 4 °C) for 15 min, and then the supernatant was desiccated in a centrifuge. The cells were re-dissolved in 100 μL water/acetonitrile (1:1, *v*/*v*) solvent for LC-MS analysis. The samples of quality control (QC) were performed by mixing 10 μL of every single sample to control the reproducibility of instrumental research.

#### 4.6.2. LC-MS/MS Analysis

A UHPLC (1290 Infinity LC, Agilent Technologies, Santa Clara, CA, USA) and a quadrupole time-of-flight (TripleTOF 6600, AB Sciex, Boston, MA, USA) were conducted for the analysis. An ACQUIY UPLC BEH column (2.1 mm × 100 mm, 1.7 µm, Waters, Milford, MA, USA) was used to analyze the samples for separation. Then, 25 mM ammonium hydroxide and 25 mM ammonium acetate in water (A) and acetonitrile (B) were performed as the mobile phase in both ESI positive and negative patterns. The gradient conditions were 15% A kept for 1 min, increased to 35% in 11 min, then gradually increased to 60% in 0.1 min, retained for 4 min, then reduced to 15% in 0.1 min, and, finally, a 5 min equilibration was performed.

#### 4.6.3. Statistical Data Analysis

By ProteoWizard MSConvert, the MS data was transformed from wiff.scan files to mzXML files and exported to XCMS Online (https://xcmsonline.scripps.edu, accessed on 16 May 2019). For annotation of adducts and isotopes, the Collection of Algorithms of MEtabolite pRofile Annotation (CAMERA) was used. By comparing MS/MS spectra and accuracy m/z value (<25 ppm) with an internal database built with available authentic standards, compound evaluation of metabolites was conducted. To show its contribution to the classification, we computed the VIP value of each variable in the OPLS-DA model. To measure the significance of each metabolite, metabolites in which the VIP exceeded 1.0 were further performed to Student’s *t*-test at a univariate level (*p*-values < 0.05) were regarded as statistically meaningful.

### 4.7. Bioinformatics Analysis

Gene Ontology (GO) terms were used to map the identified protein sequences to determine the biological properties and functional classification of the screened differentially abundant proteins. An NCBI BLAST search was conducted for all detected sequences with the NCBInr Medicago truncatula database for this analysis. GO analysis was performed by BLAST2GO [54]. Moreover, all differentially distinct proteins and metabolites were identified by the Kyoto Encyclopedia of Genes and Genomes (KEGG) [55]. The functional classifications and pathways (*p*-values < 0.05) were regarded as having obvious enrichment. To inquire about the enriched KEGG proteins and metabolites by an R-based software for omics results integration, the significantly changed proteins and metabolites identified between the control group and treatment group were employed, respectively.

### 4.8. qRT-PCR Analysis

The expression of genes *acnA*, *ebpS*, *clfA*, *icd*, and *gpmA* was verified by qRT-PCR. MRSA was harvested in the same requirement as the proteomics research. The measurements were carried out in triplicate. RNAprotect bacteria reagent (QIAGEN, Dusseldorf, Germany) was appended to the cells directly, to stabilize RNA. Total RNA Kit (GBCBIO Technologies, Guangzhou, China) was used to extract total RNA, and the concentration of RNA was detected by a Nanodrop 2000c spectrophotometer (Thermo Scientific, Waltham, MA, USA). qRT-PCR was conducted using a two-step method. RNA was firstly reverse-transcribed to cDNA (Vazyme, Nanjing, China); and RT-PCR was performed on QuantStudio 3 Real-Time PCR System (Applied Biosystems, Carlsbad, CA, USA) using Realtime PCR Super mix SYBRgreen with 40 cycles of denaturation for 15 s at 95 °C, annealing for 15 s at 55 °C, and extension for 42 s at 72 °C. The melting curve stage was conducted after amplification to confirm the specificity of the PCR amplification outcome. The relative quantitation was calculated by the 2^−ΔΔCT^ method [56]. The primer sequences are given in Table S6. 16S rRNA was selected as an internal reference.

### 4.9. Molecular Docking Studies

For the docking study, the program Maestro 11.8 was used to predict the possible binding behavior between Aconitate hydratase A (AcnA, UniProtKB—Q6G9K9), Isocitrate dehydrogenase (Icd, UniProtKB—Q8NW61), and actinomycin D. Because the crystal structure of AcnA and Icd had not been resolved, we first needed to find the sequence from UniProt website (https://www.uniprot.org/, accessed on 10 September 2020) and construct a tertiary structure model of them. In this study, homology modeling was conducted by SWISS-MODEL (https://swissmodel.expasy.org/, accessed on 10 September 2020), and the PDB files of the template were available from the website. The details of the template were shown in Table S7. The structure of actinomycin D was obtained from PubChem (https://pubchem.ncbi.nlm.nih.gov/, ID: 2019, accessed on 10 September 2020). All water molecules were removed, and polar hydrogen was added for the molecular docking study. Finally, the model with the best docking score was used for further analysis.

## 5. Conclusions

A marine *S. parvulus* originated actinomycin D possessed a significant inhibitory effect on MRSA. The MIC and MBC values of actinomycin to MRSA were 1 and 8 μg/mL, respectively. Proteomics integrated with metabolomics analysis revealed 261 differential proteins (up-regulated 86, down-regulated 94, new 9 and undetectable 72) and 144 differential metabolites (up-regulated 68 and down-regulated 76), and the co-mapped correlation network of omics indicated that actinomycin D induced the metabolism pathway about antibiotic sensitive in MRSA. Furthermore, qRT-PCR verified the expression of mRNA for genes *acnA* and *icd*, and the encoding proteins related to antibiotic sensitivity were predicted by molecular docking. In summary, actinomycin D might affect the antibiotic sensitivity of MRSA by disturbing the glycine, serine, and threonine metabolism and TCA cycle, also inhibiting the growth of cell wall and damaging cell membrane component. In addition, it might regulate the virulence factor SdrD, ClfA, AgrA, and LuxS to reduce virulence. These findings will contribute to a better understanding of the possible molecular mechanism of anti-MRSA actinomycin D.

## Figures and Tables

**Table 1:** Molecular docking predictive data within the ligand-target molecule couples.

Target	ΔG (kcal/mol)	Docking Score	Hydrogen Bond Location
AcnA	−48.15	−5.729	ASN445, THR444, ASN541
Icd	−26.27	−5.65	TYR333, GLU370

## Data Availability

The data presented in this study are available in Supplementary Material here.

## Supplementary Materials

The following supporting information can be downloaded at: https://www.mdpi.com/article/10.3390/md20020114/s1, Table S1: Protein quantification and analysis of significant differences, Table S2: GO annotation statistics table, Table S3: KEGG pathways in which differentially expressed proteins are involved, Table S4: Differential metabolites of leaves between the budding stage and mid-flowering stage, Table S5: Proteome-metabolome data co-analysis, Table S6: Sequences of the used primers, Table S7: The information of the template for molecular docking.
