# Supplementary material for: Molecular Details of Actinomycin D-Treated MRSA Revealed via High-Dimensional Data
Source: Mar Drugs. 2022 Jan 31;20(2):114. doi: 10.3390/md20020114 (PMC8878686; doi:10.3390/md20020114)
Supplement: Supplementary file 1 [file marinedrugs-20-00114-s001.zip › Supplementary Table S1.pdf]

Table S1. Protein quantification and analysis of significant differences.

Accession	Protein Name	Coverage	Unique Peptides	Peptides	MW [kDa]	Average-MRSA-T	Average-MRSA-CK	log_2(fold changes)[1]	Type[1]	t-test p value
Q99WJ1	Xanthine phosphoribosyltransferase	22.4	4	4	20.884	1340200000	102379666.7	13.09049	up	0.000692173
Q99VR1	7-cyano-7-deazaguanine synthase	4.1	1	1	24.887	207720000	19115650	10.866489	up	0.013270795
Q8NWQ6	Extracellular matrix-binding protein ebh	0.6	3	4	1068.5	134356666.7	12692333.33	10.585655	up	1.22982E-05
Q99TM4	UPF0337 protein SAV1625	35	2	3	6.6824	3054966667	365206666.7	8.365035	up	0.000162936
Q6GEU1	S-ribosylhomocysteine lyase	20.5	2	2	17.514	163023333.3	22159400	7.356848	up	0.000557339
Q6GI88	Regulatory protein Spx	56.5	8	8	15.44	350090000	48890333.33	7.16072	up	0.000891295
Q99W81	Transcriptional regulator CtsR	16.3	2	2	17.841	390900000	57399666.67	6.810144	up	4.69433E-05
Q99UI1	tRNA-2-methylthio-N(6)-dimethylallyladenosine synthase	8.2	5	5	58.916	85341333.33	14811233.33	5.761933	up	0.017568731
Q6GH57	Large-conductance mechanosensitive channel	5.8	1	1	13.616	41324666.67	7254533.333	5.696392	up	0.00195524
Q99WC3	Recombination protein RecR	36.9	5	5	22.072	45676000	9087233.333	5.026392	up	0.000885844
Q6GIQ3	UDP-N-acetylenolpyruvoylglucosamine reductase	11.1	4	4	33.753	151370000	34045666.67	4.446087	up	2.34085E-06
Q6GG27	50S ribosomal protein L20	35.6	6	6	13.686	1270500000	306046666.7	4.151328	up	5.35511E-05
Q6GH63	UPF0154 protein SAR1353	51.2	6	6	9.3195	2106233333	507390000	4.151113	up	0.020682308
Q6GEU7	Probable DNA-directed RNA polymerase subunit delta	55.7	8	8	20.881	618106666.7	156616666.7	3.946621	up	0.000167285
Q99U67	Probable CtpA-like serine protease	2.4	1	1	55.25	47591000	12317333.33	3.863742	up	3.70036E-06
Q6GHW1	Phosphopantetheine adenylyltransferase	6.2	1	1	18.371	57948666.67	15700000	3.690998	up	9.68537E-06
Q5HJU7	Putative surface protein SACOL0050	2.1	2	2	164.85	134264666.7	37175666.67	3.611628	up	0.030348067
Q6GJD1	50S ribosomal protein L11	62.1	9	9	14.874	6109366667	1709266667	3.574262	up	0.003355313
Q9ZHA2	Cell division protein SepF	29.9	5	5	21.023	193503333.3	55040666.67	3.515643	up	0.001181347
Q99U87	Uncharacterized hydrolase SAV1398	10.4	3	3	43.143	16129000	4660600	3.460713	up	0.002032692
Q8NW72	ATP-dependent Clp protease ATP-binding subunit ClpX	37.9	14	14	46.296	2116100000	612113333.3	3.45704	up	4.56644E-06
Q6GEV5	50S ribosomal protein L31 type B	54.8	4	4	9.7228	3253900000	954626666.7	3.408558	up	0.002409563

Q6GKT0	50S ribosomal protein L9	46.6	6	6	16.454	538246666.7	159893333.3	3.366286	up	0.004449218
Q99S23	50S ribosomal protein L23	54.9	6	6	10.605	967290000	296873333.3	3.258258	up	0.000727952
Q6GIM3	ATP-dependent Clp protease proteolytic subunit	60.5	8	8	21.513	4523133333	1393666667	3.245492	up	2.71026E-05
Q6GJJ4	Nucleoid-associated protein SAR0478	59	5	5	11.597	7416200000	2293766667	3.233197	up	0.000359587
Q6GHM6	Guanylate kinase	25.1	4	4	24.023	37709000	11833333.33	3.186676	up	0.002465052
Q2FZP6	UDP-N-acetylmuramoyl-L-alanyl-D-glutamate--L-lysine ligase	5.9	2	2	54.104	77678666.67	24557000	3.163199	up	2.66622E-05
Q99Q02	Regulatory protein MsrR	8.9	4	4	36.97	172170000	54505666.67	3.158754	up	0.001970875
Q6GH51	DNA topoisomerase 4 subunit B	11.3	8	8	74.397	539513333.3	173553333.3	3.108631	up	0.0007757
Q6GHI4	Methylenetetrahydrofolate--tRNA-(uracil-5-)-methyltransferase TrmFO	14.7	5	5	48.359	93654000	30340000	3.086816	up	0.000121195
Q6GI75	Enoyl-[acyl-carrier-protein] reductase [NADPH] FabI	14.5	4	4	27.992	834736666.7	271920000	3.069788	up	0.000407647
Q6GGD7	DNA primase	10.9	7	7	69.275	168163333.3	56380666.67	2.982642	up	2.71324E-05
Q99U78	Uncharacterized protein SAV1409	8.4	2	2	29.448	68261000	22979666.67	2.970496	up	0.002701604
Q6GGC5	30S ribosomal protein S21	48.3	5	5	6.9721	6817700000	2298633333	2.96598	up	0.001190012
Q8NVT1	ATP-dependent DNA helicase PcrA	19.6	13	13	84.073	323196666.7	109382666.7	2.954734	up	0.000914291
Q6YP15	Peptidyl-tRNA hydrolase	5.3	1	1	21.703	9316100	3202200	2.909281	up	6.73541E-05
Q99S31	50S ribosomal protein L14	59.8	9	9	13.135	4683100000	1644600000	2.847562	up	0.000650701
Q6GFD7	Probable manganese-dependent inorganic pyrophosphatase	44.7	11	11	34.068	630333333.3	222173333.3	2.837124	up	0.022946612
Q6GFF6	Aspartyl/glutamyl-tRNA(Asn/Gln) amidotransferase subunit C	85	6	6	11.267	1658733333	588330000	2.819393	up	3.10898E-05
Q6GH52	Glycerol-3-phosphate acyltransferase	6.9	1	1	22.232	15624000	5572433.333	2.803802	up	0.001187114
Q99UM1	DNA topoisomerase 1	15.5	10	10	79.07	96731333.33	34739666.67	2.784463	up	0.0002295
Q99SH6	DEAD-box ATP-dependent RNA helicase CshA	23.9	11	11	56.942	589653333.3	213440000	2.762619	up	0.000596421
Q6G8P5	Translation initiation factor IF-3	24.6	4	4	20.213	1320866667	486253333.3	2.716417	up	7.62608E-05
Q6GIG3	Lipoyl synthase	37.7	11	11	34.885	1220233333	451746666.7	2.701145	up	7.85131E-06

Q99VB5	Chaperone protein ClpB	31.9	23	25	98.329	1322900000	500403333.3	2.643667	up	0.000386096
Q6GH50	DNA topoisomerase 4 subunit A	8.9	6	6	91.077	264900000	100826333.3	2.62729	up	0.011554133
Q6GCA6	Single-stranded DNA-binding protein 2	38.9	6	6	18.539	2010200000	774730000	2.59471	up	0.000249078
Q6GJV1	30S ribosomal protein S18	56.2	5	5	9.3098	513116666.7	198840000	2.580551	up	0.000905252
P66083	50S ribosomal protein L19	54.3	1	9	13.376	11094733.33	4327633.333	2.563695	up	0.009428881
Q6GBU9	50S ribosomal protein L1	63.5	12	12	24.708	2728333333	1068100000	2.55438	up	0.000162713
Q99S28	50S ribosomal protein L16	34.7	5	5	16.242	3032266667	1196683333	2.533892	up	0.036318853
Q6GKU1	DNA replication and repair protein RecF	3.8	1	1	42.415	2294800	911486.6667	2.517645	up	0.002768185
Q99S51	50S ribosomal protein L13	81.4	13	13	16.333	4178633333	1698466667	2.460239	up	0.000120011
Q6G9B3	Holliday junction resolvase RecU	14.4	3	3	24.443	28424333.33	11576000	2.455454	up	0.00814617
Q99VT5	HTH-type transcriptional regulator MgrA	56.5	8	8	17.089	881373333.3	360593333.3	2.444231	up	1.26916E-05
Q6GEJ1	50S ribosomal protein L29	50.7	4	4	8.0902	3643000000	1500700000	2.427534	up	0.000107996
Q931Q3	50S ribosomal protein L27	34	3	3	10.315	2636633333	1090666667	2.417451	up	0.000384784
Q99S26	50S ribosomal protein L22	65.8	8	8	12.835	3413666667	1419933333	2.404103	up	0.00063021
Q99TK6	50S ribosomal protein L21	66.7	7	7	11.333	2268400000	946550000	2.396493	up	0.000749004
Q6GJD2	Transcription termination/antitermination protein NusG	54.9	10	10	20.663	1802500000	757096666.7	2.380806	up	0.000250339
Q99WT9	ESAT-6 secretion machinery protein EssC	1.8	3	3	170.93	238406666.7	100319333.3	2.376478	up	0.009531204
Q6GF42	10 kDa chaperonin	45.7	5	5	10.416	2714366667	1158066667	2.343878	up	0.001457008
Q99S46	50S ribosomal protein L17	45.9	6	6	13.748	374046666.7	165186666.7	2.264388	up	0.000212003
Q6GD89	Chromosomal replication initiator protein DnaA	32.7	15	15	51.965	487370000	217466666.7	2.241125	up	0.000852467
Q6GF14	3-isopropylmalate dehydratase large subunit	6.2	3	3	50.254	297073333.3	133176333.3	2.230677	up	0.03584097
Q8NX05	50S ribosomal protein L19	54.3	1	9	13.362	2413233333	1085300000	2.223563	up	0.000435787
Q6GJC3	30S ribosomal protein S12	47.4	8	8	15.287	6456433333	2915933333	2.214191	up	0.006142404
Q6GHF0	Protein RecA	41.2	12	12	37.656	856346666.7	388203333.3	2.205923	up	0.000241235
Q8NY74	Uncharacterized protein MW0361	55	15	15	35.612	986386666.7	447400000	2.204709	up	0.002578834
Q59803	Chorismate synthase	5.9	2	2	43.059	51373666.67	23688333.33	2.168733	up	0.025739491
Q6GJC6	DNA-directed RNA polymerase subunit beta	59.3	58	58	133.2	7325100000	3386366667	2.163115	up	8.67609E-06
Q8NWA9	Protein GrpE	57.2	2	9	24.008	303950000	140790000	2.158889	up	0.017360473

Q99VD4	Putative peptidyl-prolyl cis-trans isomerase	53.3	7	7	21.619	417673333.3	196390000	2.126755	up	0.001919681
Q6GIA4	3-oxoacyl-[acyl-carrier-protein] synthase 3	9.9	2	2	33.879	80925666.67	38084000	2.124926	up	0.01355387
Q931G5	30S ribosomal protein S10	57.8	5	5	11.579	1411733333	668866666.7	2.110635	up	0.000929709
Q8NWZ0	tRNA pseudouridine synthase B	17	5	5	34.534	380643333.3	181180000	2.100913	up	0.004450787
Q6GEI3	50S ribosomal protein L3	36.8	6	6	23.718	1900100000	905006666.7	2.099543	up	0.003493191
Q8NWF2	Segregation and condensation protein B	3.9	1	1	20.205	11662666.67	5607333.333	2.079895	up	0.003523539
Q6GEK1	50S ribosomal protein L30	83.1	7	7	6.5536	4377000000	2113000000	2.071462	up	0.002808692
Q6GHL1	50S ribosomal protein L28	35.5	3	3	6.9771	770153333.3	372060000	2.069971	up	0.0071421
Q6GG08	ATP-dependent 6-phosphofructokinase	33.5	9	9	34.823	382856666.7	186113333.3	2.057116	up	4.81066E-05
Q6GJV3	30S ribosomal protein S6	67.3	9	9	11.595	1467400000	715943333.3	2.049604	up	0.001238955
Q6GBU7	50S ribosomal protein L7/L12	90.2	1	11	12.711	13802000000	6757200000	2.042562	up	0.0008945
P19529	Replication initiation protein	6.4	1	2	38.163	12222666.67	5991066.667	2.040149	up	0.000881638
Q99WA2	50S ribosomal protein L25	34.6	6	6	23.787	2290933333	1124633333	2.037049	up	0.001163532
Q99VW2	Response regulator protein GraR	5.8	1	1	26.066	3067700	-[2]	-	up	-
Q6GG87	Putative pre-16S rRNA nuclease	12.7	2	2	15.865	2888333	-	-	up	-
Q8NXZ2	Dihydropteroate synthase	6.4	1	1	29.522	4144800	-	-	up	-
Q6GH72	Catalase	69.9	1	26	58.377	2248747	-	-	up	-
Q9KIN5	Undecaprenyl-diphosphatase	4.1	1	1	32.312	7786767	-	-	up	-
P41368	Isoleucine--tRNA ligase	1.8	2	2	118.87	10601867	-	-	up	-
Q6GI15	Phosphoribosylformylglycinamidine synthase subunit PurL	1.5	1	1	79.501	9301167	-	-	up	-
Q99UQ0	Probable dual-specificity RNA methyltransferase RlmN	4.4	1	1	41.904	12973333	-	-	up	-
P39857	Capsular polysaccharide biosynthesis glycosyltransferase CapH	3.7	1	1	41.41	19175333	-	-	up	-
Q6GII3	Glycine cleavage system H protein	46	4	4	14.08	609890000	1223233333	0.498588	down	0.008959431
Q93P61	PTS system glucose-specific EIIA component	29.5	4	4	17.96	482346666.7	968390000	0.498091	down	0.000516869
Q6GHD5	Glycerol kinase	38.2	17	17	55.667	515090000	1040606667	0.49499	down	0.000209123
Q6GFM3	Uroporphyrinogen decarboxylase	22	6	6	39.294	48985333.33	99244333.33	0.493583	down	0.007019228

Q8NXH0	Probable cysteine desulfurase	21.1	6	6	46.276	74806333.33	152416666.7	0.490802	down	8.64147E-05
Q6GHY9	Dihydrolipoyl dehydrogenase	60.7	25	25	49.451	2201266667	4497800000	0.48941	down	0.000313118
Q6GA10	Carbamoyl-phosphate synthase large chain	11.3	12	12	117.18	65547666.67	142293333.3	0.460652	down	0.005546586
Q6GK73	L-lactate dehydrogenase 1	14.5	4	4	34.565	1005836667	2275133333	0.4421	down	0.002580152
Q6GEE4	Urease subunit alpha	11	5	5	61.775	238766666.7	541036666.7	0.441313	down	0.000353555
Q8NX25	Orotate phosphoribosyltransferase	3.9	1	1	22.015	10978966.67	25867333.33	0.424434	down	0.008033638
P99174	Deoxyribose-phosphate aldolase 2	53.2	2	9	23.341	375716666.7	906343333.3	0.414541	down	0.003647871
Q99T33	UPF0342 protein SAV1845	73.7	8	8	13.31	1982633333	4816266667	0.411654	down	0.028622851
Q7A4R9	Response regulator protein VraR	39.2	7	7	23.559	53669333.33	132790000	0.404167	down	0.00051981
Q8NW95	5-oxoprolinase subunit A	4.4	2	2	27.421	54117000	135839666.7	0.398389	down	0.033983073
Q99QV3	Lactonase drp35	6.2	2	2	35.963	4507500	11545033.33	0.390428	down	0.002463711
Q6GJR7	Alkyl hydroperoxide reductase C	68.8	11	11	20.976	1915500000	4929133333	0.388608	down	0.003382539
Q6GJF0	Pyridoxal 5-phosphate synthase subunit PdxS	66.8	15	15	31.992	1097566667	2842233333	0.386163	down	0.000371641
Q6GJB8	Q8NVE6	63.5	1	21	42.847	174406666.7	459713333.3	0.379381	down	0.000752799
Q99SC3	Pyrimidine-nucleoside phosphorylase	61.2	20	20	46.366	335300000	884893333.3	0.378916	down	0.00079422
Q8NXY3	Putative pyridoxal phosphate-dependent acyltransferase	68.9	2	22	42.891	1388733333	3759700000	0.369373	down	0.00017854
Q99V36	Probable quinol oxidase subunit 2	48.9	15	15	41.777	596743333.3	1619700000	0.368428	down	0.004505389
Q6GHQ6	Ribosomal RNA small subunit methyltransferase H	18.6	4	4	35.681	14910666.67	41673666.67	0.357796	down	0.000973029
Q99TF3	Putative universal stress protein SAV1710	79.5	10	10	18.475	874793333.3	2452433333	0.356704	down	0.001322045
Q99WW8	Ribitol-5-phosphate cytidylyltransferase 1	68.5	9	12	26.656	59463666.67	167440000	0.355134	down	0.009930546
Q6G8N8	Dephospho-CoA kinase	39.6	6	6	23.623	5764650	16576666.67	0.347757	down	0.021774976
Q7A522	Putative dipeptidase SA1572	52	16	16	52.824	118096666.7	350860000	0.336592	down	0.000256149
Q6GGU2	Nucleoside diphosphate kinase	29.5	4	4	16.575	88633333.33	266553333.3	0.332516	down	0.002896198
Q99VH8	Organic hydroperoxide resistance protein-like	25.7	3	3	15.339	237943333.3	722143333.3	0.329496	down	0.001488111
Q931H9	Accessory gene regulator A	14.7	4	4	27.921	42709333.33	130060000	0.328382	down	0.00031095
Q99SF4	ATP synthase gamma chain	20.1	4	4	32.106	29757333.33	90687000	0.328132	down	0.002002733

Q5HF24	D-alanine aminotransferase	55.7	11	11	31.893	115673333.3	370823333.3	0.311937	down	0.02343008
Q8NVI5	Serine-protein kinase RsbW	54.1	8	8	17.92	42354666.67	139533333.3	0.303545	down	0.000701602
Q6G9K9	Aconitate hydratase A	42.6	28	28	98.968	958636666.7	3200800000	0.299499	down	1.65729E-05
Q8NW75	Glutamate-1-semialdehyde 2,1-aminomutase 1	18	6	6	46.388	214710000	739343333.3	0.290406	down	0.003836199
Q99U34	3-5 exonuclease DinG	2.2	2	2	104.22	4062000	14339666.67	0.28327	down	0.0020004
Q8NX95	Bifunctional protein FolD	60.5	3	14	30.843	115310000	407193333.3	0.283182	down	3.0411E-05
Q6G859	Uncharacterized protein SAS1797	52.6	6	6	18.632	637730000	2323500000	0.27447	down	0.016716348
Q8NVA1	Molybdopterin molybdenumtransferase	18.4	7	7	44.989	12627666.67	46073000	0.27408	down	0.000279477
Q6GBT3	Probable branched-chain-amino-acid aminotransferase	38.3	2	9	40.086	66480000	244293333.3	0.272132	down	2.4178E-05
Q6GHJ0	Succinate--CoA ligase [ADP-forming] subunit beta	59	21	21	42.086	1883600000	7039166667	0.267588	down	0.000271538
Q6GFW8	UDP-N-acetylmuramate--L-alanine ligase	10.3	5	5	49.202	46171666.67	177263333.3	0.260469	down	0.000137265
Q2YU22	Glutamate-1-semialdehyde 2,1-aminomutase 2	20.3	1	7	46.775	10855133.33	42861333.33	0.253262	down	0.003579885
Q99VG5	UPF0337 protein SAV0840	75	8	9	7.0186	3083233333	12241600000	0.251865	down	0.011261972
Q8NVE6	Glutamine--fructose-6-phosphate aminotransferase [isomerizing]	40.9	16	16	65.849	2134033333	8685833333	0.245691	down	0.000109984
Q8NXY2	Protein/nucleic acid deglycase HchA	24.7	7	7	32.18	78827333.33	328170000	0.240203	down	0.001489452
Q8NWM8	30S ribosomal protein S1	73.7	2	25	43.287	607776666.7	2553566667	0.238011	down	0.000259657
Q8NV80	Putative 2-hydroxyacid dehydrogenase MW2224	66.2	15	15	34.674	147750000	633370000	0.233276	down	6.22603E-06
Q6GIF4	D-alanyl carrier protein	43.6	3	3	9.0631	38517800	170920000	0.225356	down	0.008970923
Q5HJK3	Putative aldehyde dehydrogenase AldA	59.2	22	22	53.658	120856666.7	542116666.7	0.222935	down	0.0006867
Q9EZ10	2,3,4,5-tetrahydropyridine-2,6-dicarboxylate N-acetyltransferase	16.7	4	4	25.258	39250333.33	178496666.7	0.219894	down	0.000411146
Q99RW4	Putative formate dehydrogenase SA2102	23.2	3	19	111.29	137293333.3	634403333.3	0.216413	down	0.003267046
Q6GDS2	D-lactate dehydrogenase	32.4	10	10	36.755	36728666.67	178446666.7	0.205824	down	0.012478183

Q6GEP7	Alkaline shock protein 23	74.6	11	11	19.191	731810000	3708300000	0.197344	down	0.001616808
Q5HCU6	Fructose-bisphosphate aldolase class 1	82.8	24	24	33.054	616066666.7	3197066667	0.192697	down	0.000429403
Q7A7E0	N-acetylmuramoyl-L-alanine amidase sle1	38.9	3	8	35.835	99301333.33	533573333.3	0.186106	down	0.001083896
Q8NX01	Succinate--CoA ligase [ADP-forming] subunit alpha	65.9	14	14	31.526	481310000	2699766667	0.178278	down	0.00239102
Q99W56	Uncharacterized epimerase/dehydratase SAV0553	26.5	1	6	36.052	7095350	40214333.33	0.176438	down	0.031229892
Q8NW61	Isocitrate dehydrogenase [NADP]	39.3	14	14	46.436	315260000	1884500000	0.167291	down	0.00025468
Q931R8	2-oxoglutarate dehydrogenase E1 component	18.9	13	13	105.36	23972000	145896666.7	0.164308	down	0.001266425
Q5HJY7	Serine--tRNA ligase	62.4	25	25	48.639	256790000	1570700000	0.163488	down	0.00523062
Q5HES4	Fumarate hydratase class II	31.5	12	12	51.107	138683333.3	874646666.7	0.158559	down	0.000169899
A0A0H3JVA1	Poly(ribitol-phosphate) beta-N-acetylglucosaminyltransferase TarS	13.8	6	6	66.254	9078366.667	59462666.67	0.152673	down	1.76107E-05
Q99VM3	Ribosome hibernation promotion factor	38.4	7	7	22.213	75111000	510226666.7	0.147211	down	2.62844E-05
O86488	Serine-aspartate repeat-containing protein D	24.9	23	24	142.77	31921666.67	233536666.7	0.136688	down	0.000997388
Q8NYD0	Pseudouridine-5-phosphate glycosidase	39.4	10	10	32.809	13303000	97596000	0.136307	down	0.000949765
Q5HEK9	NH(3)-dependent NAD(+) synthetase	62.6	11	11	30.696	41588333.33	314410000	0.132274	down	0.001526923
Q99WU4	ESAT-6 secretion system extracellular protein A	71.1	7	7	11.036	22037200	167870000	0.131275	down	0.014430419
Q99W73	Cysteine--tRNA ligase	27.7	11	11	53.684	10643766.67	81112666.67	0.131222	down	0.038558628
Q5HH71	Putative phosphoesterase SACOL1020	49.7	1	8	19.326	37506333.33	286050000	0.131118	down	0.022697414
Q8NWR7	Dihydrolipoyllysine-residue succinyltransferase component of 2-oxoglutarate dehydrogenase complex	40.8	3	14	46.673	141373333.3	1156633333	0.122228	down	5.63921E-05
Q99VD0	NAD-specific glutamate dehydrogenase	53.4	17	17	45.76	95673333.33	798636666.7	0.119796	down	5.06289E-07
Q820A6	Pyruvate dehydrogenase E1 component subunit alpha	40	12	12	41.382	296253333.3	2557500000	0.115837	down	0.013166555
Q6G6Q5	2,3-bisphosphoglycerate-dependent phosphoglycerate mutase	67.1	13	13	26.68	67996666.67	601353333.3	0.113073	down	0.002190588
Q99UU0	Carbamate kinase 1	33.5	9	9	33.596	10396000	94843000	0.109613	down	3.84191E-05

Q99TF4	Alanine dehydrogenase 2	28	8	8	40.079	34651333.33	316136666.7	0.109609	down	2.09723E-06
Q2YYT1	Putative formate dehydrogenase SAB2186c	22	2	18	111.31	12466500	115343333.3	0.108082	down	0.000303244
Q99S93	UPF0457 protein SA1975.1	75.6	7	7	10.006	470420000	4366966667	0.107722	down	6.32792E-06
Q6GAW9	Ornithine aminotransferase 2	46.2	16	16	43.417	117966666.7	1234033333	0.095594	down	0.00015795
Q5HF67	Uncharacterized peptidase SACOL1756	22.2	5	5	39.553	23740000	314003333.3	0.075604	down	6.47064E-06
Q2G296	Formate--tetrahydrofolate ligase	47	1	20	59.856	146720000	1963533333	0.074722	down	0.000152706
Q6GHU0	Thioredoxin	81.7	7	7	11.44	821826666.7	13609333333	0.060387	down	0.000896326
Q8NWD0	Probable glycine dehydrogenase (decarboxylating) subunit 2	35.7	13	13	54.781	24691000	427220000	0.057795	down	3.06741E-06
Q8NY72	UPF0355 protein MW0363	84.4	8	8	15.122	18158150	325173333.3	0.055841	down	0.034937621
Q8NUR2	1-pyrroline-5-carboxylate dehydrogenase	60.9	26	26	56.868	173283333.3	3366133333	0.051478	down	0.000171541
Q99VC0	Coenzyme A disulfide reductase	42.9	13	13	49.29	5198966.667	124230000	0.04185	down	4.48897E-05
Q99WZ7	Formate acetyltransferase	64.5	40	40	84.861	98426333.33	3240200000	0.030377	down	0.007892112
Q8NUM9	L-lactate dehydrogenase 2	55.5	12	12	34.42	73664566.67	2953733333	0.024939	down	0.006656171
Q8NUK7	Arginine deiminase	85.4	33	33	46.914	54613666.67	2663700000	0.020503	down	0.001428148
Q8NUK8	Ornithine carbamoyltransferase, catabolic	82.4	18	19	37.762	52790350	3135700000	0.016835	down	0.001590626
Q8NWC9	Probable glycine dehydrogenase (decarboxylating) subunit 1	22.5	9	9	49.716	2487433.333	157120000	0.015831	down	6.70456E-09
Q6GG19	Glyceraldehyde-3-phosphate dehydrogenase 2	30.2	9	9	37.003	18748766.67	1938166667	0.009673	down	6.17739E-06
A0A0H3K686	Immunoglobulin G-binding protein A	58.1	26	26	55.554	36428333.33	4161733333	0.008753	down	0.000532659
Q8NX96	Bifunctional autolysin	72.8	0	75	137.41	63677333.33	16119000000	0.00395	down	0.010927187
Q6GKH2	Superoxide dismutase [Mn/Fe] 2	53.8	8	9	23.041	586176.6667	162063333.3	0.003617	down	0.00022189
A0A0H3JT43	Glycine cleavage system H-like protein	31.8	2	2	12.422	-	17110000	-	down	-
Q99X11	FMN-dependentNADH-azoreductase	20.7	3	3	23.352	-	30989667	-	down	-

Q8NY94	5-methyltetrahydropteroyltriglutamate--homocysteinemethyltransferase	5.8	3	4	85.074	-	3959833	-	down	-
Q6GBR8	Glucosamine-6-phosphatedeaminase	17.9	3	3	28.467	-	11643033	-	down	-
Q6GIF6	D-alanine--D-alanylcarrierproteinligase	19	7	7	54.585	-	49043000	-	down	-
Q8NXF2	Argininosuccinatesynthase	44.1	1	14	44.441	-	8292700	-	down	-
Q99UX4	Iron-regulatedsurfacedeterminantproteinA	3.4	1	1	38.745	-	17828333	-	down	-
Q8NWZ3	RibosomematurationfactorRimP	9.7	1	1	17.627	-	3924233	-	down	-
Q6GH71	50SribosomalproteinL332	26.5	1	1	5.9318	-	15073500	-	down	-
Q9EZ12	4-hydroxy-tetrahydrodipicolinatesynthase	8.5	2	2	32.584	-	20387500	-	down	-
Q99U66	UPF0346proteinSAV1421	17.8	1	1	8.8697	-	4777450	-	down	-
Q6G943	Exodeoxyribonuclease7smallsubunit	77.6	4	4	8.7596	-	86649000	-	down	-
Q6G8Y8	ChaperoneproteinDnaJ	19	6	6	41.76	-	54601667	-	down	-
Q99TQ0	5-methylthioadenosine/S-adenosylhomocysteinenucleosidase	5.7	1	1	24.534	-	3750133	-	down	-
Q6GG71	D-aminoacyl-tRNAdeacylase	22.7	3	3	16.725	-	10114200	-	down	-
Q8NW56	UPF0173metal-dependenthydrolaseMW1650	17.5	2	2	25.25	-	6674933	-	down	-
Q8NVU3	RegulatoryproteinRecX	5.1	1	1	32.241	-	3153167	-	down	-
Q6GFE5	UPF0316proteinSAR2004	9	1	1	22.955	-	1829700	-	down	-
Q6G7Q4	Dihydroxy-aciddehydratase	7.1	3	3	60.021	-	6244000	-	down	-
Q8NVI9	3-isopropylmalatedehydratasesmallsubunit	10.5	2	2	21.605	-	7459067	-	down	-
Q6GEX3	ATPsynthaseepsilonchain	11.9	1	1	14.844	-	81636333	-	down	-
Q99SE9	ATPsynthasesubunita	2.9	1	1	27.63	-	1740700	-	down	-
Q99SE4	UPF0340proteinSAV2114	10.3	2	2	18.895	-	2342550	-	down	-
Q6GEJ4	50SribosomalproteinL24	16.2	2	2	11.536	-	25914333	-	down	-
Q8NV65	Imidazolonepropionase	7	3	3	45.052	-	24735333	-	down	-
Q6GEA1	Formimidoylglutamase	3.9	1	1	34.436	-	7294467	-	down	-
Q99RL2	Immunoglobulin-bindingproteinSbi	28.7	11	11	50.042	-	366556667	-	down	-

Q8NUM0	Oxygen-dependentcholinedehydrogenase	14.6	8	8	63.623	-	24697667	-	down	-
Q99R06	HTH-typetranscriptionalregulatorArcR	32.1	6	6	27.428	-	34348000	-	down	-
Q6GDC8	Imidazoleglycerol-phosphatedehydratase	18.8	3	3	21.46	-	16760333	-	down	-
Q99X56	Hemeoxygenase(staphylobilin-producing)2	52.8	5	5	12.791	-	112355000	-	down	-
Q5HJB7	Iron-sulfurclusterrepairproteinScdA	35.7	2	5	25.485	-	54188667	-	down	-
Q5HGX8	UPF0637proteinSACOL1115	34.3	5	5	24.019	-	59989667	-	down	-
Q8NX50	Fibrinogen-bindingprotein	10.9	2	2	18.764	-	16346667	-	down	-
Q5HFU2	Elastin-bindingproteinEbpS	24.7	7	7	53.221	-	64858000	-	down	-
Q5HF42	Formate--tetrahydrofolateligase	46.8	1	20	59.913	-	6481767	-	down	-
Q6G6A5	ProbabletransglycosylaseIsaA	36.1	4	4	24.203	-	71485333	-	down	-
Q8NUI2	1-(5-phosphoribosyl)-5-[(5-phosphoribosylamino)methylideneamino]imidazole-4-carboxamideisomerase	11.1	2	2	26.09	-	5461567	-	down	-
Q6GAW1	SignalpeptidaseIB	20.4	4	4	21.692	-	9372350	-	down	-
Q6GH44	PeptidemethioninesulfoxidereductaseMsrA1	6.5	1	1	19.611	-	3500250	-	down	-
Q6G9D7	PeptidemethioninesulfoxidereductaseMsrA2	29.4	4	4	20.588	-	43445333	-	down	-
Q99SN8	Uncharacterizedleukocidin-likeprotein1	7.1	2	2	38.662	-	6082533	-	down	-
Q6GA63	dITP/XTPpyrophosphatase	7.2	1	1	21.403	-	3508400	-	down	-
Q931E9	PutativesurfaceproteinSAV2496/SAV2497	7	3	3	122.21	-	37391000	-	down	-
Q6GHD0	GlutathioneperoxidasehomologBsaA	12.7	2	2	18.117	-	46808500	-	down	-
P64356	HistidinebiosynthesisbifunctionalproteinHisIE	5.2	1	1	23.944	-	117180000	-	down	-
Q6GK63	Ribitol-5-phosphatecytidylyltransferase2	26.9	4	7	26.533	-	33656667	-	down	-
Q8NUI5	Lipase1	1	1	1	76.675	-	27763333	-	down	-
Q6GHN4	Dihydroorotase	7.1	2	2	46.385	-	15155667	-	down	-
Q6GKH9	Diacetylreductase[(S)-acetoinforming]	32.6	5	5	27.215	-	33564000	-	down	-
Q931G1	Urocanatehydratase	18.6	9	9	60.663	-	233460000	-	down	-

Q9LAB5	ImmunodominantstaphylococcalantigenB	25.1	4	4	19.37	-	139615000	-	down	-
Q99RB2	PutativeNAD(P)HnitroreductaseSAV2523	9.4	2	2	25.374	-	7989067	-	down	-
Q5HD73	UncharacterizedoxidoreductaseSACOL2488	44.2	7	7	24.604	-	64692333	-	down	-
Q99RQ9	UncharacterizedlipoproteinSAV2368	15.7	2	2	22.837	-	33198000	-	down	-
Q99RT8	LysostaphinresistanceproteinA	4.5	1	1	46.785	-	20438333	-	down	-
Q9F0R1	HTH-typetranscriptionalregulatorSarR	46.1	5	5	13.669	-	107142333	-	down	-
Q99SJ1	L-threoninedehydratasebiosyntheticIlvA	3.8	2	2	46.97	-	13732667	-	down	-
Q5HEH9	Uncharacterizedleukocidin-likeprotein2	19.1	6	6	40.434	-	93543667	-	down	-
Q7A4W3	SignaltransductionproteinTRAP	32.3	5	5	19.547	-	111703333	-	down	-
Q99V38	Probablequinoloxidasesubunit3	5	1	1	23.057	-	29637333	-	down	-
Q99W49	FMN-dependentNADPH-azoreductase	28.7	4	4	20.911	-	15948333	-	down	-
Q8NY95	Probableacetyl-CoAacyltransferase	20.1	4	4	41.717	-	33157333	-	down	-
Q99X00	UncharacterizedresponseregulatoryproteinSAV0223	12.7	2	2	29.575	-	4020650	-	down	-
Q99X30	N-acetylmuramicacid6-phosphateetherase	3.7	1	1	32.382	-	8518067	-	down	-
Q2FWX9	4,4-diaponeurosporen-aldehydedehydrogenase	9.8	3	3	51.741	-	6788450	-	down	-
Q2G015	ClumpingfactorA	1.3	1	1	96.446	-	30428333	-	down	-
Q2G1B9	Ribulose-5-phosphatereductase1	13.8	3	3	38.451	-	12879667	-	down	-
Q2G222	N-acetylmuramoyl-L-alanineamidasedomain-containingproteinSAOUHSC_02979	8.2	4	4	69.252	-	7827033	-	down	-
Q99UT4	UncharacterizedN-acetyltransferaseSA1019	23.3	3	3	17.002	-	19546333	-	down	-
Q2YY06	Dihydrolipoyllysine-residuesuccinyltransferasecomponentof2-oxoglutaratedehydrogenasecomplex	33.9	0	12	46.705	-	56901000	-	down	-
Q5HH31	Bifunctionalautolysin	71.7	0	74	137.33	-	325340000	-	down	-

A0A0H3JT43	GlycinecleavagesystemH-likeprotein	31.8	2	2	12.422	-	17110000	-	down	-
Q99X11	FMN-dependentNADH-azoreductase	20.7	3	3	23.352	-	30989667	-	down	-

[1]up and down represent up-regulation and down-regulation respectively. [2] The symbol ”-” indicates that there is no value.
