# Supplementary material for: Molecular Details of Actinomycin D-Treated MRSA Revealed via High-Dimensional Data
Source: Mar Drugs. 2022 Jan 31;20(2):114. doi: 10.3390/md20020114 (PMC8878686; doi:10.3390/md20020114)
Supplement: Supplementary file 1 [file marinedrugs-20-00114-s001.zip › Supplementary Table S3.pdf]

Table S3. KEGG pathways in which differentially expressed proteins are involved.

Map_ID	Map Name	Test	TestAll	Ref	RefAll	P value	FDR	RichFactor
ko05150	Staphylococcus aureus infection	12	261	14	623	0.000892	0.098122	0.857142857
ko00660	C5-Branched dibasic acid metabolism	4	261	4	623	0.030393	0.650899	1
ko00310	Lysine degradation	7	261	9	623	0.031913	0.650899	0.777777778
ko00720	Carbon fixation pathways in prokaryotes	9	261	13	623	0.042154	0.650899	0.692307692
ko00630	Glyoxylate and dicarboxylate metabolism	9	261	13	623	0.042154	0.650899	0.692307692
ko00260	Glycine, serine and threonine metabolism	9	261	13	623	0.042154	0.650899	0.692307692
ko00280	Valine, leucine and isoleucine degradation	5	261	6	623	0.049576	0.650899	0.833333333
ko00270	Cysteine and methionine metabolism	6	261	8	623	0.061371	0.650899	0.75
ko00340	Histidine metabolism	8	261	12	623	0.072956	0.650899	0.666666667
ko00650	Butanoate metabolism	3	261	3	623	0.073037	0.650899	1
ko01503	Cationic antimicrobial peptide (CAMP) resistance	3	261	3	623	0.073037	0.650899	1
ko00020	Citrate cycle (TCA cycle)	10	261	16	623	0.076465	0.650899	0.625
ko03010	Ribosome	29	261	56	623	0.076924	0.650899	0.517857143
ko00290	Valine, leucine and isoleucine biosynthesis	5	261	7	623	0.114447	0.839277	0.714285714
ko00380	Tryptophan metabolism	5	261	7	623	0.114447	0.839277	0.714285714
ko00640	Propanoate metabolism	7	261	11	623	0.122352	0.841169	0.636363636
ko02026	Biofilm formation - Escherichia coli	2	261	2	623	0.17512	0.853398	1
ko00040	Pentose and glucuronate interconversions	3	261	4	623	0.20097	0.853398	0.75
ko00330	Arginine and proline metabolism	5	261	8	623	0.202907	0.853398	0.625
ko04922	Glucagon signaling pathway	4	261	6	623	0.205316	0.853398	0.666666667
ko03440	Homologous recombination	4	261	6	623	0.205316	0.853398	0.666666667
ko00561	Glycerolipid metabolism	5	261	9	623	0.306182	0.853398	0.555555556
ko00770	Pantothenate and CoA biosynthesis	4	261	7	623	0.326475	0.853398	0.571428571
ko02020	Two-component system	8	261	16	623	0.33778	0.853398	0.5
ko00071	Fatty acid degradation	3	261	5	623	0.35019	0.853398	0.6
ko00473	D-Alanine metabolism	3	261	5	623	0.35019	0.853398	0.6
ko04213	Longevity regulating pathway - multiple species	3	261	5	623	0.35019	0.853398	0.6
ko05230	Central carbon metabolism in cancer	3	261	5	623	0.35019	0.853398	0.6

ko04146	Peroxisome	3	261	5	623	0.35019	0.853398	0.6
ko00300	Lysine biosynthesis	3	261	5	623	0.35019	0.853398	0.6
ko00620	Pyruvate metabolism	11	261	23	623	0.351769	0.853398	0.47826087
ko00480	Glutathione metabolism	2	261	3	623	0.379285	0.853398	0.666666667
ko00981	Insect hormone biosynthesis	2	261	3	623	0.379285	0.853398	0.666666667
ko00410	beta-Alanine metabolism	2	261	3	623	0.379285	0.853398	0.666666667
ko05111	Biofilm formation - Vibrio cholerae	2	261	3	623	0.379285	0.853398	0.666666667
ko00053	Ascorbate and aldarate metabolism	2	261	3	623	0.379285	0.853398	0.666666667
ko00903	Limonene and pinene degradation	2	261	3	623	0.379285	0.853398	0.666666667
ko04016	MAPK signaling pathway - plant	2	261	3	623	0.379285	0.853398	0.666666667
ko05418	Fluid shear stress and atherosclerosis	2	261	3	623	0.379285	0.853398	0.666666667
ko00072	Synthesis and degradation of ketone bodies	1	261	1	623	0.418941	0.853398	1
ko04934	Cushing syndrome	1	261	1	623	0.418941	0.853398	1
ko00966	Glucosinolate biosynthesis	1	261	1	623	0.418941	0.853398	1
ko00590	Arachidonic acid metabolism	1	261	1	623	0.418941	0.853398	1
ko04621	NOD-like receptor signaling pathway	1	261	1	623	0.418941	0.853398	1
ko04931	Insulin resistance	1	261	1	623	0.418941	0.853398	1
ko04152	AMPK signaling pathway	1	261	1	623	0.418941	0.853398	1
ko03320	PPAR signaling pathway	1	261	1	623	0.418941	0.853398	1
ko00472	D-Arginine and D-ornithine metabolism	1	261	1	623	0.418941	0.853398	1
ko05200	Pathways in cancer	1	261	1	623	0.418941	0.853398	1
ko05120	Epithelial cell signaling in Helicobacter pylori infection	1	261	1	623	0.418941	0.853398	1
ko04918	Thyroid hormone synthesis	1	261	1	623	0.418941	0.853398	1
ko05211	Renal cell carcinoma	1	261	1	623	0.418941	0.853398	1
ko04626	Plant-pathogen interaction	1	261	1	623	0.418941	0.853398	1
ko04214	Apoptosis - fly	1	261	1	623	0.418941	0.853398	1
ko04211	Longevity regulating pathway	2	261	4	623	0.557601	0.927389	0.5
ko00450	Selenocompound metabolism	2	261	4	623	0.557601	0.927389	0.5
ko00625	Chloroalkane and chloroalkene degradation	2	261	4	623	0.557601	0.927389	0.5
ko04068	FoxO signaling pathway	2	261	4	623	0.557601	0.927389	0.5

ko00860	Porphyrin and chlorophyll metabolism	4	261	9	623	0.565133	0.927389	0.444444444
ko00250	Alanine, aspartate and glutamate metabolism	6	261	14	623	0.573296	0.927389	0.428571429
ko00190	Oxidative phosphorylation	6	261	14	623	0.573296	0.927389	0.428571429
ko00220	Arginine biosynthesis	6	261	14	623	0.573296	0.927389	0.428571429
ko02024	Quorum sensing	3	261	7	623	0.621507	0.927389	0.428571429
ko04112	Cell cycle - Caulobacter	3	261	7	623	0.621507	0.927389	0.428571429
ko00790	Folate biosynthesis	3	261	7	623	0.621507	0.927389	0.428571429
ko04212	Longevity regulating pathway - worm	3	261	7	623	0.621507	0.927389	0.428571429
ko05014	Amyotrophic lateral sclerosis (ALS)	1	261	2	623	0.662761	0.927389	0.5
ko04011	MAPK signaling pathway - yeast	1	261	2	623	0.662761	0.927389	0.5
ko05010	Alzheimer disease	1	261	2	623	0.662761	0.927389	0.5
ko00360	Phenylalanine metabolism	1	261	2	623	0.662761	0.927389	0.5
ko00362	Benzoate degradation	1	261	2	623	0.662761	0.927389	0.5
ko04013	MAPK signaling pathway - fly	1	261	2	623	0.662761	0.927389	0.5
ko00760	Nicotinate and nicotinamide metabolism	1	261	2	623	0.662761	0.927389	0.5
ko00261	Monobactam biosynthesis	1	261	2	623	0.662761	0.927389	0.5
ko05016	Huntington disease	1	261	2	623	0.662761	0.927389	0.5
ko00791	Atrazine degradation	1	261	2	623	0.662761	0.927389	0.5
ko00900	Terpenoid backbone biosynthesis	1	261	2	623	0.662761	0.927389	0.5
ko00010	Glycolysis / Gluconeogenesis	11	261	28	623	0.682084	0.927389	0.392857143
ko04066	HIF-1 signaling pathway	2	261	5	623	0.695875	0.927389	0.4
ko00910	Nitrogen metabolism	2	261	5	623	0.695875	0.927389	0.4
ko00430	Taurine and hypotaurine metabolism	2	261	5	623	0.695875	0.927389	0.4
ko03020	RNA polymerase	2	261	5	623	0.695875	0.927389	0.4
ko00680	Methane metabolism	5	261	13	623	0.699757	0.927389	0.384615385
ko00670	One carbon pool by folate	3	261	8	623	0.724384	0.937439	0.375
ko00195	Photosynthesis	3	261	8	623	0.724384	0.937439	0.375
ko03030	DNA replication	2	261	6	623	0.79628	0.951558	0.333333333
ko03430	Mismatch repair	3	261	9	623	0.80401	0.951558	0.333333333
ko00750	Vitamin B6 metabolism	1	261	3	623	0.804499	0.951558	0.333333333

ko03060	Protein export	1	261	3	623	0.804499	0.951558	0.333333333
ko00785	Lipoic acid metabolism	1	261	3	623	0.804499	0.951558	0.333333333
ko00333	Prodigiosin biosynthesis	1	261	3	623	0.804499	0.951558	0.333333333
ko00471	D-Glutamine and D-glutamate metabolism	1	261	3	623	0.804499	0.951558	0.333333333
ko00400	Phenylalanine, tyrosine and tryptophan biosynthesis	1	261	3	623	0.804499	0.951558	0.333333333
ko00550	Peptidoglycan biosynthesis	4	261	12	623	0.815203	0.953961	0.333333333
ko00051	Fructose and mannose metabolism	2	261	7	623	0.866189	0.99123	0.285714286
ko00520	Amino sugar and nucleotide sugar metabolism	5	261	16	623	0.872036	0.99123	0.3125
ko00052	Galactose metabolism	1	261	4	623	0.886799	0.99123	0.25
ko00780	Biotin metabolism	1	261	4	623	0.886799	0.99123	0.25
ko00240	Pyrimidine metabolism	8	261	25	623	0.892107	0.99123	0.32
ko00983	Drug metabolism - other enzymes	1	261	5	623	0.93453	0.999299	0.2
ko00500	Starch and sucrose metabolism	1	261	5	623	0.93453	0.999299	0.2
ko03018	RNA degradation	2	261	9	623	0.944728	0.999299	0.222222222
ko00061	Fatty acid biosynthesis	2	261	9	623	0.944728	0.999299	0.222222222
ko03420	Nucleotide excision repair	1	261	6	623	0.96218	0.999299	0.166666667
ko02060	Phosphotransferase system (PTS)	1	261	6	623	0.96218	0.999299	0.166666667
ko00710	Carbon fixation in photosynthetic organisms	2	261	10	623	0.965068	0.999299	0.2
ko00030	Pentose phosphate pathway	3	261	14	623	0.972139	0.999299	0.214285714
ko00230	Purine metabolism	8	261	31	623	0.982092	0.999299	0.258064516
ko00564	Glycerophospholipid metabolism	1	261	9	623	0.992761	0.999299	0.111111111
ko00970	Aminoacyl-tRNA biosynthesis	4	261	26	623	0.999299	0.999299	0.153846154
