# Supplementary material for: Molecular Details of Actinomycin D-Treated MRSA Revealed via High-Dimensional Data
Source: Mar Drugs. 2022 Jan 31;20(2):114. doi: 10.3390/md20020114 (PMC8878686; doi:10.3390/md20020114)
Supplement: Supplementary file 1 [file marinedrugs-20-00114-s001.zip › Supplementary Table S4.pdf]

Table S4. Differential metabolites of leaves between the budding stage and mid-flowering stage.

Ionization mode	Metabolite no.	Adduct	RT(s)	m/z	Metabolite	VIP	Fold-change	P-value
ESI(+)	1	(M+H)+	827.766	231.09688	Pro-Asp	1.716521002	0.031725183	2.56669E-06
ESI(+)	2	(M+H)+	795.713	786.17382	Flavin adenine dinucleotide (FAD)	1.716756556	0.039892782	6.0925E-06
ESI(+)	3	(M+H-2H2O)+	759.971	242.11265	Arg-Cys	1.825266514	0.040696834	1.11933E-06
ESI(+)	4	(M+H-2H2O)+	780.429	270.14412	Arg-Met	2.280503892	0.044029022	4.15581E-07
ESI(+)	5	(M+CH3CN+H)+	781.466	326.14514	His-Glu	1.009719626	0.053455303	1.48423E-07
ESI(+)	6	(M+CH3CN+Na)+	684.808	457.21063	(-)-Tylocrebrine	1.322731506	0.055354548	5.13557E-05
ESI(+)	7	(M+H)+	970.74	272.171	Pro-Arg	2.922418314	0.056835838	2.32903E-09
ESI(+)	8	(M+H)+	816.847	245.11275	Pro-Glu	1.480345427	0.060185482	2.07523E-06
ESI(+)	9	(M+CH3CN+H)+	584.541	320.15999	Pro-Tyr	2.4096227	0.085069586	3.93207E-05
ESI(+)	10	(M+H)+	918.799	234.14427	Lys-Ser	1.012932707	0.087696057	1.39632E-07
ESI(+)	11	(M+H)+	1094.47	130.08521	.beta.-Homoproline	1.581729474	0.089548111	1.00767E-08
ESI(+)	12	(M+CH3CN+Na)+	843.688	213.12245	Triethanolamine	1.052678872	0.094879462	6.43086E-05
ESI(+)	13	(M+H)+	1021.375	290.14516	Asp-Arg	6.874206078	0.100501042	2.16355E-08
ESI(+)	14	(M+CH3CN+H)+	715.892	244.1286	Pro-Ser	6.219000254	0.111135584	0.000161459
ESI(+)	15	(M+H)+	543.105	220.11711	Pantothenate	1.916615826	0.119925617	4.11178E-07
ESI(+)	16	(M+H)+	888.573	262.14984	Ser-Arg	1.213775378	0.120424115	0.004048488
ESI(+)	17	(M+H-H2O)+	63.434	120.08003	Tyramine	1.133162373	0.1233688	1.68546E-06
ESI(+)	18	(M+H)+	677.702	187.10701	Pro-Ala	5.443999023	0.128610851	3.65765E-09
ESI(+)	19	(M+H)+	861.904	227.11321	L-Carnosine	1.378423734	0.131994722	3.06254E-09
ESI(+)	20	(M+H)+	920.071	262.13912	Lys-Asp	1.213480582	0.140717404	0.000673525
ESI(+)	21	(M+CH3CN+H)+	827.097	501.22918	5-Methyltetrahydrofolate (5-Methyl-THF)	1.221575518	0.144459256	4.48089E-05
ESI(+)	22	(M+H)+	739.98	132.06451	Hydroxyproline	1.254757734	0.146616972	0.000338875
ESI(+)	23	(M+H)+	835.875	191.06547	Gly-Asp	1.099901936	0.169355452	5.796E-07
ESI(+)	24	(M+H)+	735.947	244.12861	Pro-Gln	2.253273911	0.175251268	1.86228E-06
ESI(+)	25	(M+H)+	436.2485	124.0384	Nicotinate	1.407095398	0.178045788	5.46719E-06
ESI(+)	26	(M+H-H2O)+	177.248	115.08562	Ornithine	1.040781647	0.19115274	6.57653E-06
ESI(+)	27	(M+H)+	664.234	217.11774	Pro-Thr	2.160646225	0.193703018	6.15903E-08

ESI(+)	28	(M+H)+	701.644	173.09124	Glycylproline	1.937665338	0.204678211	1.39703E-06
ESI(+)	29	(M+H)+	96.0085	123.05464	Nicotinamide	2.183507385	0.210714061	7.84717E-09
ESI(+)	30	(M+H)+	874.791	664.11536	Nicotinamide adenine dinucleotide (NAD)	5.52930936	0.212634181	4.36973E-05
ESI(+)	31	(M+H)+	697.352	288.20254	Leu-Arg	1.826820361	0.22236366	3.17259E-06
ESI(+)	32	(M+H)+	1088.33	203.14985	NG,NG-dimethyl-L-arginine(ADMA)	1.112770305	0.238828054	0.002676914
ESI(+)	33	(M+NH4)+	956.2135	247.13952	Pro-Asn	1.652213604	0.240845596	0.00088118
ESI(+)	34	(M+H)+	248.239	150.07676	N6-Methyladenine	1.656474172	0.264621556	1.92231E-06
ESI(+)	35	(M+H-2H2O)+	876.5	524.05723	ADP-ribose	1.18032651	0.304245841	0.003164266
ESI(+)	36	(M+H)+	848.0895	348.06976	Adenosine monophosphate (AMP)	2.897826277	0.335096644	0.000401712
ESI(+)	37	(M+H)+	346.5665	136.06078	Adenine	1.001988337	0.434830207	0.000508165
ESI(+)	38	(M+H)+	634.6685	116.06983	D-Proline	1.989415056	0.538096152	1.9565E-06
ESI(+)	39	(M+H)+	782.4635	148.05966	L-Glutamate	1.319732367	0.611924236	6.44035E-05
ESI(+)	40	(M+H)+	90.45	104.06994	3-Aminobutanoic acid	1.699589341	0.736383282	0.016621898
ESI(+)	41	(M+H)+	567.34	132.10104	L-Isoleucine	2.634120975	1.388065517	0.030731169
ESI(+)	42	(M+H-H2O)+	461.6915	195.08697	Gly-His	1.61614558	1.418884468	0.016916984
ESI(+)	43	(M+H-H2O)+	304.132	251.14986	His-Ile	3.511497265	1.453859682	0.048946434
ESI(+)	44	(M+H-H2O)+	290.91	235.1187	His-Pro	8.623945777	1.476714247	0.007030112
ESI(+)	45	(M+H)+	890.618	277.13899	L-Saccharopine	1.096060889	1.52784196	0.016460024
ESI(+)	46	(M+H-H2O)+	421.4865	209.10287	His-Ala	1.040000525	1.540863934	0.007718382
ESI(+)	47	(M+H)+	824.709	205.08132	Gly-Glu	1.291268819	1.598849074	0.003553831
ESI(+)	48	(M+H-H2O)+	278.316	251.10236	Tyr-Ser	1.324616397	1.719664833	0.029745081
ESI(+)	49	(M+CH3CN+H)+	58.1055	354.33568	Phytanic acid	1.166813745	1.750769883	0.016627909
ESI(+)	50	(M-2H+3Na)+	911.3045	364.06488	2'-O-methylguanosine	1.843839476	1.777974041	0.035697605
ESI(+)	51	(M+H)+	346.11	114.06537	Creatinine	2.553412712	1.812658317	0.027690856
ESI(+)	52	(M+H-H2O)+	332.0935	237.13407	His-Val	2.271120867	1.932644915	0.000698315
ESI(+)	53	(2M+H)+	76.1265	509.45536	cis-9-Palmitoleic acid	5.321507935	1.951589206	0.00214067
ESI(+)	54	(M+H)+	627.355	130.08523	D-Pipecolinic acid	1.061266795	1.983015875	0.000350709
ESI(+)	55	(M+H)+	518.466	215.13826	Pro-Val	1.073585399	2.178968468	0.012724043
ESI(+)	56	(M+H)+	652.9445	118.08538	Betaine	1.559896661	2.240896077	0.011497012
ESI(+)	57	(M+H-H2O)+	310.493	285.13421	His-Phe	2.059367834	2.318092168	0.00797533

ESI(+)	58	(M+H)+	289.013	252.10832	Deoxyadenosine	1.03456887	2.617156147	0.016033706
ESI(+)	59	(M+H-H2O)+	640.421	158.09174	L-Citrulline	1.27889112	2.775298448	0.00129445
ESI(+)	60	(M+H)+	1071.68	399.1438	S-Adenosylmethionine	1.552351422	3.395707889	0.043618833
ESI(+)	61	(M+H)+	619.8235	189.08608	N-Acetylglutamine	1.416042266	3.551181528	0.000111985
ESI(+)	62	M+	831.9315	265.11092	Thiamine	4.175995731	5.09237414	0.000176487
ESI(+)	63	(M+H)+	736.727	175.10732	N2-Acetyl-L-ornithine	1.001008383	5.145493307	0.00295759
ESI(+)	64	(M+H)+	593.806	130.04868	L-Pyroglutamic acid	2.045909049	5.506508991	3.96309E-05
ESI(+)	65	(M+H)+	326.421	245.07647	Uridine	1.251544246	5.798356844	0.005808544
ESI(+)	66	(M+H)+	966.146	405.00866	Uridine 5'-diphosphate (UDP)	1.039013867	6.196180117	0.014497894
ESI(+)	67	(M+H)+	327.109	113.03372	Uracil	1.947268954	6.47768979	0.004836631
ESI(+)	68	(M+H)+	119.883	170.08066	Pyridoxine	1.065747255	6.492769309	0.001894997
ESI(+)	69	(M+H)+	737.673	120.06449	L-Threonine	1.465749975	7.355947197	0.000679389
ESI(+)	70	(M+H)+	592.8085	138.05417	Anthranilic acid (Vitamin L1)	1.393688476	7.97108792	3.70833E-05
ESI(+)	71	(M+H)+	263.7905	282.1193	N6-methyladenosine	2.581359024	8.188226956	0.000299134
ESI(+)	72	(M+H)+	1199.89	175.11836	L-Arginine	5.242125949	9.660411738	0.000310995
ESI(+)	73	(M+H)+	730.272	162.11154	L-Carnitine	4.209990771	13.10221699	0.000180515
ESI(+)	74	(M+H)+	761.554	217.12856	N-.alpha.-Acetyl-L-arginine	2.425414253	13.94027006	3.07612E-08
ESI(+)	75	(M+H)+	684.808	145.13234	N-Acetylcadaverine	2.092242476	15.00018696	7.27832E-08
ESI(+)	76	(M+H)+	455.616	269.0877	Inosine	1.088935179	19.70852235	0.000510328
ESI(-)	77	(M-H)-	125.9235	271.22732	16-Hydroxypalmitic acid	3.123414324	0.030765043	3.67034E-06
ESI(-)	78	(M-H)-	617.9725	187.07188	alpha-N-Acetyl-L-glutamine	2.909206328	0.046968508	1.51656E-06
ESI(-)	79	(M-H)-	460.718	601.35543	Garcinol	1.106452205	0.052572498	0.000268672
ESI(-)	80	(M-H)-	814.3735	243.02746	L-Fucose-1-phosphate	1.442327486	0.054215425	9.19669E-06
ESI(-)	81	(M-H)-	330.6005	103.03949	2-hydroxy-butanoic acid	1.479435777	0.059338482	0.000224256
ESI(-)	82	(2M-H)-	59.742	375.27418	3-Hydroxycapric acid	1.623295922	0.078411678	1.44569E-06
ESI(-)	83	(M-H)-	682.8535	171.07715	Glycylproline	1.972096536	0.079981179	1.01502E-05
ESI(-)	84	(M-H)-	681.9105	130.05068	Hydroxyproline	1.951231219	0.087351294	0.007397003
ESI(-)	85	(M-H)-	68.468	325.31081	Heneicosanoic acid	2.169945221	0.094112554	1.14085E-10
ESI(-)	86	(M-H)-	852.785	363.03405	Xanthylic acid (XMP)	1.043251821	0.110732215	5.02369E-05
ESI(-)	87	(M-H)-	487.9255	218.1033	Pantothenate	4.910225524	0.117448943	8.16272E-08

ESI(-)	88	(M-H)-	67.847	301.21671	Eicosapentaenoic acid	1.74691917	0.12895033	0.005613187
ESI(-)	89	(M-H)-	67.865	337.31045	Erucic acid	2.179456536	0.147472342	2.17153E-07
ESI(-)	90	(M-H)-	575.169	114.05575	D-Proline	8.871497513	0.155911578	3.77689E-07
ESI(-)	91	(M-H)-	373.7495	158.08191	Acetyl-DL-Valine	1.027760718	0.156963074	3.09486E-06
ESI(-)	92	(M-H)-	70.206	297.27944	Pristanic acid	2.948246006	0.179431552	4.51139E-07
ESI(-)	93	(M-H)-	383.583	122.0243	Nicotinate	1.723660581	0.183931785	3.44154E-06
ESI(-)	94	(M-H)-	808.232	540.0525	Cyclic adenosine diphosphate ribose	4.495479518	0.227346157	1.86224E-05
ESI(-)	95	(M-H)-	785.1925	346.05517	Adenosine monophosphate (AMP)	4.051010116	0.229792922	4.33533E-05
ESI(-)	96	(M-H)-	800.369	225.09898	L-Carnosine	1.149952203	0.261357449	7.46039E-08
ESI(-)	97	(M-H)-	89.081	269.24807	Heptadecanoic acid	1.094628206	0.285553966	7.50641E-06
ESI(-)	98	(M-H)-	690.313	117.01909	Succinate	5.395767605	0.300392593	1.88838E-08
ESI(-)	99	(M-H)-	690.324	73.0292	Propionic acid	1.686411249	0.31587453	3.84719E-08
ESI(-)	100	(M-H)-	185.4695	87.04497	Isobutyric acid	2.343837797	0.342768509	2.42408E-06
ESI(-)	101	(M-H)-	287.941	134.04681	Adenine	2.737928698	0.369057186	1.68915E-05
ESI(-)	102	(M-H)-	582.3905	159.07734	D-Alanyl-D-alanine (D-Ala-D-Ala)	4.175401458	0.376679901	1.49669E-05
ESI(-)	103	(M-H)-	115.109	241.07275	Lumichrome	1.524338616	0.397148092	9.96745E-06
ESI(-)	104	(M-H)-	725.086	146.04586	L-Glutamate	9.777879175	0.424307657	1.60006E-06
ESI(-)	105	(M-H)-	69.0565	311.2948	Arachidic acid	1.16543757	0.426842976	6.1442E-07
ESI(-)	106	(M-H)-	524.554	148.04331	L-Methionine	1.519369162	0.441094438	0.000392926
ESI(-)	107	(M-H)-	73.6825	255.23256	Palmitic acid	2.397439412	0.610830828	4.51651E-06
ESI(-)	108	(M-H)-	71.336	283.26394	Stearic acid	3.313678384	0.862514813	0.047329527
ESI(-)	109	(M-H2O-H)-	739.8315	141.01681	2-Oxoadipic acid	11.49174404	1.112807543	0.015515897
ESI(-)	110	(M-H)-	152.832	111.01986	Uracil	10.51210199	1.226014774	0.00067318
ESI(-)	111	(M-H)-	75.996	227.20136	Myristic acid	2.803482855	1.276702086	0.014404169
ESI(-)	112	(M-H)-	504.632	130.08721	L-Leucine	6.895815098	1.398479662	0.000752565
ESI(-)	113	(M-H2O-H)-	493.522	139.02572	Allantoin	1.441240374	1.429235003	0.043449459
ESI(-)	114	(M-H)-	69.026	303.23246	Arachidonic Acid (peroxide free)	1.584200502	1.552145445	0.031914029
ESI(-)	115	(M-H)-	848.6815	362.04999	Guanosine 5'-monophosphate (GMP)	1.848374725	1.660465656	0.020949487
ESI(-)	116	(M-H)-	555.074	116.07139	L-Valine	4.196935031	1.789289279	0.008828941
ESI(-)	117	(M-H)-	789.191	171.00631	Glycerol 3-phosphate	1.952111201	1.840893433	0.001039034

ESI(-)	118	(M-H)-	455.134	130.05049	N-Acetyl-L-alanine	1.100378506	1.943116692	0.002826663
ESI(-)	119	(M-H)-	77.1415	213.18577	Tridecanoic acid (Tridecylic acid)	1.801870073	2.176208104	3.45535E-06
ESI(-)	120	(M-H)-	71.9395	279.23241	Linoleic acid	2.413834103	2.418586118	4.239E-05
ESI(-)	121	(M-H)-	676.278	173.093	N2-Acetyl-L-ornithine	1.582431484	2.645710147	0.004892409
ESI(-)	122	(M-H)-	535.628	128.03524	L-Pyroglutamic acid	6.29720086	2.713615472	0.000350794
ESI(-)	123	(M-H)-	783.4335	173.00883	cis-Aconitate	1.048118796	2.776346524	2.4079E-06
ESI(-)	124	(M-H)-	73.1145	277.21673	all cis-(6,9,12)-Linolenic acid	1.14535822	2.872880202	0.000854398
ESI(-)	125	(M-H)-	659.711	118.05048	L-Threonine	3.488117562	3.071740942	1.342E-05
ESI(-)	126	(M-H)-	873.988	191.01935	Citrate	4.570144981	3.751829848	0.000261101
ESI(-)	127	(M-H)-	784.0595	678.09484	UDP-N-acetylmuraminate	1.486460615	3.756361339	4.88909E-06
ESI(-)	128	(M-H)-	709.672	104.03481	L-Serine	1.259364456	4.117379151	2.1847E-07
ESI(-)	129	(M+CH3COO)-	940.39	368.9986	D-Ribulose 1,5-bisphosphate	1.215117147	4.84016868	6.17194E-05
ESI(-)	130	(M-H)-	554.7165	105.01882	Glyceric acid	1.400768531	5.135071914	0.000346204
ESI(-)	131	(M-H)-	676.452	245.11398	gamma-L-Glutamyl-L-valine	1.451866518	5.672909573	2.72153E-06
ESI(-)	132	(M-H)-	475.32	283.0687	Xanthosine	1.754776292	6.838975681	0.004185335
ESI(-)	133	M-	836.887	261.03789	O-Phosphotyrosine	3.04304649	7.096500564	6.57612E-06
ESI(-)	134	(M-H)-	291.8	243.06179	Uridine	2.231817557	7.6442517	0.002585684
ESI(-)	135	(M-H)-	791.08	565.04627	Uridine diphosphate glucose(UDP-D-Glucose)	1.244101384	8.065073667	3.39963E-06
ESI(-)	136	(M-H)-	201.709	131.07111	Hydroxyisocaproic acid	8.559323435	8.201841199	2.67769E-05
ESI(-)	137	(M-H)-	906.724	402.99366	Uridine 5'-diphosphate (UDP)	2.032542646	8.728524688	0.001513626
ESI(-)	138	(M-H)-	537.799	116.03484	Acetylglycine	1.390001614	9.38506194	2.6507E-06
ESI(-)	139	(M-H)-	48.8015	319.23036	20-HETE	3.32027569	10.63714649	1.69114E-07
ESI(-)	140	(M-H)-	905.6735	184.9854	DL-2-Phosphoglycerate	1.70814079	11.248415	7.82654E-05
ESI(-)	141	(2M-H)-	190.818	295.13935	Mevalonic acid	1.599473999	18.84960198	0.000523089
ESI(-)	142	(M+CH3COO)-	833.272	289.03265	D-Ribose 5-phosphate	1.931262155	19.64274604	3.66552E-05
ESI(-)	143	(M-H)-	403.589	267.07291	Inosine	2.994709886	24.4095334	0.000841881
ESI(-)	144	(M-H)-	674.959	276.98815	2-C-Methyl-D-erythritol 2,4-cyclodiphosphate	1.707182055	54.94563652	1.27879E-06
