# Supplementary material for: Molecular Details of Actinomycin D-Treated MRSA Revealed via High-Dimensional Data
Source: Mar Drugs. 2022 Jan 31;20(2):114. doi: 10.3390/md20020114 (PMC8878686; doi:10.3390/md20020114)
Supplement: Supplementary file 1 [file marinedrugs-20-00114-s001.zip › Supplementary Table S5.pdf]

Table S5. Proteome-metabolome data co-analysis.

ID	Name	#Cpd	Cpd	
			Metabolites	Proteins（accession）
ko00230	Purine metabolism	18	Adenine丨D-Ribose 5-phosphate丨Adenosine monophosphate (AMP)丨Xanthylic acid (XMP)丨Inosine丨Xanthosine丨Guanosine 5'-monophosphate (GMP)丨Hypoxanthine丨ADP-ribose丨Deoxyadenosine	Q99WJ1丨Q6GEU7丨Q6GHM6丨Q6GJC6丨Q6GEE4丨Q6GGU2丨Q99UU0丨Q6GA63
ko00630	Glyoxylate and dicarboxylate metabolism	17	Succinate丨L-Serine丨L-Glutamate丨cis-Aconitate丨D-Ribulose 1,5-bisphosphate丨DL-2-Phosphoglycerate丨Citrate丨Glyceric acid	Q6GII3丨Q6GHY9丨Q6G9K9丨Q99RW4丨Q2YYT1丨Q8NWD0丨Q8NWC9丨Q6GH72丨Q8NY95
ko00260	Glycine, serine and threonine metabolism	15	L-Serine丨L-Threonine丨DL-2-Phosphoglycerate丨Glyceric acid丨Betaine丨Choline	Q6GII3丨Q6GHY9丨Q6GJB8丨Q8NXY3丨Q6G6Q5丨Q8NWD0丨Q8NWC9丨Q8NUM0丨Q99SJ1
ko05230	Central carbon metabolism in cancer	14	Succinate丨L-Serine丨L-Glutamate丨DL-2-Phosphoglycerate丨Citrate丨L-Methionine丨L-Leucine丨L-Valine丨L-Histidine丨L-Arginine丨L-Isoleucine	Q6GG08丨Q820A6丨Q6G6Q5
ko00020	Citrate cycle (TCA cycle)	13	Succinate丨cis-Aconitate丨Citrate	Q6GHY9丨Q6G9K9丨Q6GHJ0丨Q8NX01丨Q8NW61丨Q931R8丨Q5HES4丨Q8NWR7丨Q820A6丨Q2YY06
ko00720	Carbon fixation pathways in prokaryotes	13	Succinate丨cis-Aconitate丨Citrate丨5-Methyltetrahydrofolate (5-Methyl-THF)	Q6G9K9丨Q8NX95丨Q6GHJ0丨Q8NX01丨Q8NW61丨Q5HES4丨Q2G296丨Q5HF42丨Q8NY95
ko00240	Pyrimidine metabolism	13	Uridine diphosphate glucose(UDP-D-Glucose)丨Uracil丨Uridine 5'-diphosphate (UDP)丨Uridine丨Uridine 5'-monophosphate (UMP)	Q6GEU7丨Q6GJC6丨Q6GA10丨Q8NX25丨Q99SC3丨Q6GGU2丨Q8NYD0丨Q6GHN4
ko00970	Aminoacyl-tRNA biosynthesis	13	L-Serine丨L-Glutamate丨L-Threonine丨L-Methionine丨L-Leucine丨L-Valine丨L-Histidine丨L-Arginine丨L-Isoleucine	Q6GFF6丨Q5HJY7丨Q99W73丨P41368
ko00620	Pyruvate metabolism	12	Succinate	Q6GHY9丨Q6GK73丨Q8NXY2丨Q5HJK3丨Q6GDS2丨Q5HES4丨Q820A6丨Q99WZ7丨Q8NUM9丨Q8NY95丨Q2FWX9
ko00010	Glycolysis / Gluconeogenesis	12	DL-2-Phosphoglycerate	Q6GG08丨Q93P61丨Q6GHY9丨Q6GK73丨Q5HJK3丨Q5HCU6丨Q820A6丨Q6G6Q5丨Q8NUM9丨Q6GG19丨Q2FWX9

ko02020	Two-component system	11	Succinate丨L-Glutamate丨Citrate	Q6GD89丨Q7A4R9丨Q931H9丨Q6GIF4丨Q99VW2丨Q6GIF6丨Q8NY95丨Q99X00
ko00340	Histidine metabolism	11	L-Carnosine丨L-Glutamate丨L-Histidine	Q5HJK3丨Q8NV65丨Q6GEA1丨Q6GDC8丨Q8NUI2丨P64356丨Q931G1丨Q2FWX9
ko00330	Arginine and proline metabolism	11	D-Proline丨L-Glutamate丨Hydroxyproline丨L-Arginine丨Creatinine丨S-Adenosylmethionine	Q5HF24丨Q5HJK3丨Q6GAW9丨Q8NUR2丨Q2FWX9
ko00310	Lysine degradation	10	Succinate丨2-Oxoadipic acid丨L-Saccharopine	Q5HF24丨Q5HJK3丨Q931R8丨Q8NWR7丨Q8NY95丨Q2FWX9丨Q2YY06
ko00270	Cysteine and methionine metabolism	10	L-Serine丨L-Methionine丨S-Methyl-5'-thioadenosine丨S-Adenosylmethionine	Q6GEU1丨Q6GK73丨Q6GBT3丨Q8NUM9丨Q8NY94丨Q99TQ0
ko00220	Arginine biosynthesis	10	L-Glutamate丨N2-Acetyl-L-ornithine丨L-Arginine丨L-Citrulline	Q6GEE4丨Q99VD0丨Q99UU0丨Q8NUK7丨Q8NUK8丨Q8NXF2
ko00680	Methane metabolism	10	L-Serine丨DL-2-Phosphoglycerate丨Glyceric acid丨2-Oxoadipic acid丨Tyramine	Q6GG08丨Q99RW4丨Q5HCU6丨Q6G6Q5丨Q2YYT1
ko00640	Propanoate metabolism	9	Succinate丨Propionic acid	Q6GHY9丨Q6GK73丨Q6GHJ0丨Q8NX01丨Q99WZ7丨Q8NUM9丨Q8NY95
ko00250	Alanine, aspartate and glutamate metabolism	9	Succinate丨L-Glutamate丨Citrate	Q6GA10丨Q8NVE6丨Q99VD0丨Q99TF4丨Q8NUR2丨Q8NXF2
ko00561	Glycerolipid metabolism	9	Uridine diphosphate glucose(UDP-D-Glucose)丨DL-2-Phosphoglycerate丨Glyceric acid丨Glycerol 3-phosphate	Q6GH52丨Q6GHD5丨Q5HJK3丨Q8NUI5丨Q2FWX9
ko00290	Valine, leucine and isoleucine biosynthesis	9	L-Threonine丨L-Leucine丨L-Valine丨L-Isoleucine	Q6GF14丨Q6GBT3丨Q6G7Q4丨Q8NVI9丨Q99SJ1
ko00190	Oxidative phosphorylation	8	Succinate丨Nicotinamide adenine dinucleotide (NAD)	Q6GFD7丨Q99V36丨Q99SF4丨Q6GEX3丨Q99SE9丨Q99V38
ko00520	Amino sugar and nucleotide sugar metabolism	8	Uridine diphosphate glucose(UDP-D-Glucose)丨L-Fucose-1-phosphate丨N-Acetylmannosamine	Q6GIQ3丨Q93P61丨Q8NVE6丨Q6GBR8丨Q99X30
ko00280	Valine, leucine and isoleucine degradation	8	L-Leucine丨L-Valine丨L-Isoleucine	Q6GHY9丨Q6GBT3丨Q5HJK3丨Q8NY95丨Q2FWX9

ko00380	Tryptophan metabolism	7	2-Oxoadipic acid丨Anthranilic acid (Vitamin L1)	Q5HJK3丨Q931R8丨Q6GH72丨Q8NY95丨Q2FWX9
ko00770	Pantothenate and CoA biosynthesis	7	Pantothenate丨Uracil丨L-Valine	Q6GHW1丨Q6G8N8丨Q6GBT3丨Q6G7Q4
ko04922	Glucagon signaling pathway	7	Succinate丨DL-2-Phosphoglycerate丨Citrate	Q6GK73丨Q820A6丨Q6G6Q5丨Q8NUM9
ko00660	C5-Branched dibasic acid metabolism	6	L-Glutamate丨cis-Aconitate	Q6GF14丨Q6GHJ0丨Q8NX01丨Q8NVI9
ko00860	Porphyrin and chlorophyll metabolism	6	L-Glutamate丨L-Threonine	Q6GFM3丨Q8NW75丨Q2YU22丨Q99X56
ko00030	Pentose phosphate pathway	6	D-Ribose 5-phosphate丨DL-2-Phosphoglycerate丨Glyceric acid	Q6GG08丨P99174丨Q5HCU6
ko00410	beta-Alanine metabolism	6	L-Carnosine丨Pantothenate丨Uracil丨L-Histidine	Q5HJK3丨Q2FWX9
ko00061	Fatty acid biosynthesis	6	Palmitic acid丨Myristic acid丨Stearic acid丨cis-9-Palmitoleic acid	Q6GI75丨Q6GIA4
ko00760	Nicotinate and nicotinamide metabolism	6	Succinate丨Propionic acid丨Nicotinate丨Nicotinamide丨Nicotinamide adenine dinucleotide (NAD)	Q5HEK9
ko00550	Peptidoglycan biosynthesis	5	D-Alanyl-D-alanine (D-Ala-D-Ala)	Q6GIQ3丨Q2FZP6丨Q6GFW8丨Q9KIN5
ko04212	Longevity regulating pathway - worm	5	Nicotinamide丨Nicotinamide adenine dinucleotide (NAD)	Q6GIM3丨Q6GKH2丨Q6GH72
ko00300	Lysine biosynthesis	5	2-Oxoadipic acid丨L-Saccharopine	Q7A522丨Q9EZ10丨Q9EZ12
ko00650	Butanoate metabolism	5	Succinate丨L-Glutamate	Q99WZ7丨Q6GKH9丨Q8NY95
ko00480	Glutathione metabolism	5	L-Glutamate丨gamma-L-Glutamyl-L-valine丨L-Pyroglutamic acid	Q8NW61丨Q6GHD0
ko00966	Glucosinolate biosynthesis	5	L-Methionine丨L-Leucine丨L-Valine丨L-Isoleucine	Q6GF14
ko00261	Monobactam biosynthesis	5	L-Serine丨L-Threonine丨L-Arginine丨S-Adenosylmethionine	Q9EZ12
ko00040	Pentose and glucuronate interconversions	4	Uridine diphosphate glucose(UDP-D-Glucose)	Q99WW8丨Q6GK63丨Q2G1B9
ko00670	One carbon pool by folate	4	5-Methyltetrahydrofolate (5-Methyl-THF)	Q8NX95丨Q2G296丨Q5HF42
ko00473	D-Alanine metabolism	4	D-Alanyl-D-alanine (D-Ala-D-Ala)	Q5HF24丨Q6GIF4丨Q6GIF6
ko00071	Fatty acid degradation	4	Palmitic acid	Q5HJK3丨Q8NY95丨Q2FWX9
ko00710	Carbon fixation in photosynthetic organisms	4	D-Ribose 5-phosphate丨D-Ribulose 1,5-bisphosphate	Q5HCU6丨Q6GG19

ko04211	Longevity regulating pathway	4	Adenosine monophosphate (AMP)丨Nicotinamide adenine dinucleotide (NAD)	Q6GKH2丨Q6GH72
ko04068	FoxO signaling pathway	4	L-Glutamate丨Adenosine monophosphate (AMP)	Q6GKH2丨Q6GH72
ko00564	Glycerophospholipid metabolism	4	Glycerol 3-phosphate丨Triethanolamine丨Choline	Q6GH52
ko00430	Taurine and hypotaurine metabolism	3	L-Glutamate	Q99VD0丨Q99TF4
ko00053	Ascorbate and aldarate metabolism	3	Uridine diphosphate glucose(UDP-D-Glucose)	Q5HJK3丨Q2FWX9
ko00910	Nitrogen metabolism	3	L-Glutamate	Q99VD0丨Q99UU0
ko00051	Fructose and mannose metabolism	3	L-Fucose-1-phosphate	Q6GG08丨Q5HCU6
ko05014	Amyotrophic lateral sclerosis (ALS)	3	L-Glutamate丨L-Arginine	Q6GH72
ko04152	AMPK signaling pathway	3	Adenosine monophosphate (AMP)丨Nicotinamide adenine dinucleotide (NAD)	Q6GG08
ko00500	Starch and sucrose metabolism	2	Uridine diphosphate glucose(UDP-D-Glucose)	Q93P61
ko00052	Galactose metabolism	2	Uridine diphosphate glucose(UDP-D-Glucose)	Q6GG08
ko00400	Phenylalanine, tyrosine and tryptophan biosynthesis	2	Anthranilic acid (Vitamin L1)	Q59803
ko04918	Thyroid hormone synthesis	2	D-Ribose 5-phosphate	Q6GHD0
ko00471	D-Glutamine and D-glutamate metabolism	2	L-Glutamate	Q6GFW8
ko00983	Drug metabolism - other enzymes	2	Nicotinamide adenine dinucleotide (NAD)	Q6GGU2
ko00472	D-Arginine and D-ornithine metabolism	2	L-Arginine	Q5HF24
ko00750	Vitamin B6 metabolism	2	Pyridoxine	Q6GJF0

ko00590	Arachidonic acid metabolism	2	Arachidonic Acid (peroxide free)	Q6GHD0
ko00900	Terpenoid backbone biosynthesis	2	Mevalonic acid	Q8NY95
ko05016	Huntington disease	2	L-Glutamate	Q6GKH2
ko00360	Phenylalanine metabolism	2	Succinate	Q5HF24
