# Supplementary material for: Molecular Details of Actinomycin D-Treated MRSA Revealed via High-Dimensional Data
Source: Mar Drugs. 2022 Jan 31;20(2):114. doi: 10.3390/md20020114 (PMC8878686; doi:10.3390/md20020114)
Supplement: Supplementary file 1 [file marinedrugs-20-00114-s001.zip › Supplementary Table S6.pdf]

Table S6. Sequences of the used primers.

Primer name	Sequence
16S rRNA-F	GCTGCCCTTTGTATTGTC
16S rRNA-R	AGATGTTGGGTTAAGTCCC
GpmA-F	CGTGCTTTAGATACAACGC
GpmA-R	CATCATAAGAACGACGCC
AcnA-F	CATCAAGTGCCTATTCGT
AcnA-R	CAGCATCAAAGATAGGCAT
Icd-F	GCGACAAACATTCGATTCCC
Icd-R	CCACTGCTTAAATGAGCCTT
ClfA-F	GCAAAATCCAGCACAACAGG
ClfA-R	TTCCTCCGCATTTGTATTGC
Ebps-F	GGTGCAGCTGGTGCAATGGGTGT
Ebps-R	GCTGCGCCTCCAGCCAAACCT
