# Supplementary material for: Molecular Details of Actinomycin D-Treated MRSA Revealed via High-Dimensional Data
Source: Mar Drugs. 2022 Jan 31;20(2):114. doi: 10.3390/md20020114 (PMC8878686; doi:10.3390/md20020114)
Supplement: Supplementary file 1 [file marinedrugs-20-00114-s001.zip › Supplementary Table S7.pdf]

Table S7. The information of the template for molecular docking.

Protein	UniProt Accession	Tamplate	Seq Identity (%)	QMEANDisCo Global[1]	Resolution (Å)
AcnA	Q6G9K9	2b3y.1.A	51.87	0.79 ± 0.05	1.85
Icd	Q8NW61	1hqs.1.A	78.62	0.83 ± 0.05	1.55

[1] QMEANDisCo Global represent an overall model quality measurement with higher numbers indicating higher quality.
